# Toward generalizable and interpretable machine learning models in healthcare: Insights from ICU outcome predictions

**DOI:** 10.1007/s10729-026-09760-y

**Published:** 2026-06-10

**Authors:** Lasse Bohlen, Julian Rosenberger, Nico Hambauer, Daniel Zähringer, Volkmar Franz, Patrick Zschech, Mathias Kraus

**Affiliations:** 1 Universität Regensburg, Bajuwarenstraße 4, Regensburg, 93053 Germany; 2 Technische Universität Dresden, Helmholtzstraße 10, Dresden, 01069 Germany; 3 Universitätsklinikum Carl Gustav Carus Dresden, Fetscherstraße 74, Dresden, 01307 Germany

**Keywords:** Healthcare, Intensive Care Units, Machine Learning, Generalizability, Interpretability

## Abstract

The application of machine learning (ML) models in healthcare management offers high potential. In particular, resource allocation and operational decision-making in intensive care units (ICUs) can benefit from ML predictions, leading to improvements in patient outcomes and operational efficiency. However, the generalizability of these models across diverse hospital settings with potentially different patient populations remains a critical challenge. This study examines the generalizability of ML-based ICU outcome prediction models built using external data. We utilize data from two sources: a European University Hospital (EUH) dataset from Universitätsklinikum Carl Gustav Carus Dresden, Germany and the Medical Information Mart for Intensive Care (MIMIC)-IV database, representing different healthcare systems and patient populations. Our approach evaluates multiple models of varying architectures and complexity across three common prediction tasks in ICU settings (mortality, length of stay, and readmission), analyzes the impact of data availability on model performance, and applies interpretability techniques to identify features and scenarios where models succeed or fail in new environments. We found that locally trained models generally outperform those using external data when sufficient local data is available. Low and medium complexity models, such as generalized additive models, demonstrate significantly superior generalizability compared to high complexity models and require substantially less local data for high-quality predictions, offering evidence-based guidance for healthcare managers dealing with limited data resources. Our results demonstrate how interpretability techniques can identify dataset differences that hinder generalizability, providing valuable insights for healthcare practitioners in implementing ML solutions across diverse hospitals. This research contributes to the development of more generalizable and interpretable ML models in healthcare.

## Highlights

- This study evaluates how well machine learning models for predicting ICU patient outcomes (mortality, length of stay, readmission) perform when transferred between different hospitals, using data from European and US healthcare systems.
- We find that simpler, more interpretable models maintain significantly better performance when transferred between hospitals compared to complex algorithms, challenging the assumption that more complex and sophisticated models are always better for healthcare applications.
- Healthcare institutions can use data availability to inform evidence-based decisions about adopting ML: external models can be useful when there is very little or no local data; low or medium complexity models tend to be preferable when there are small to medium sized datasets; and it is only when there are large locally available datasets that more complex and sophisticated models achieve superior performance.
- Our interpretability analysis provides healthcare managers with practical tools to identify which clinical features may cause external models to fail in their specific hospital setting, enabling safer model validation before implementation.

## Introduction

Intensive care units (ICUs) represent a critical and high-cost component of healthcare systems [1]. Complex decisions must be made to ensure both high-quality care and efficient resource utilization [2]. To support these decisions, effective ICU management increasingly looks towards data-driven approaches, with machine learning (ML) offering promising predictive capabilities [3, 4]. Using ML models, reliable predictions of patient outcomes can provide vital input for optimizing bed management [5], anticipating staffing needs [6], informing discharge planning [7], and improving overall operational efficiency within the ICU [8].

Following trends in the broader ML field, ML models in healthcare have become increasingly complex [9, 10]. This complexity is often justified by arguments that sophisticated architectures can better capture the multifaceted nature of patient data [11, 12]. This has led the majority of research to favor architectures such as neural networks or ensemble methods to enhance predictive accuracy [13]. However, relatively little is known about the practical requirements for successfully deploying such complex models across diverse healthcare settings. In particular, complex ML models require large amounts of well-organized, labeled, and cleaned data to find trustworthy patterns that generalize well. Unfortunately, these data requirements are primarily met only by large healthcare institutions, creating a critical gap where smaller institutions cannot develop their own sophisticated ML models [14].

As a remedy, many healthcare institutions, especially those smaller or with constrained data and computational resources, increasingly seek to leverage models developed at facilities with greater data resources [15]. However, the utility of these externally developed models depends entirely on their ability to generalize successfully, that is, to maintain satisfactory performance when applied to distinct patient populations beyond the initial cohort used during development [16]. This quality becomes crucial when predictive models are deployed across different hospitals or healthcare systems, where variations in patient demographics, clinical practices, and data collection procedures can lead to inaccurate predictions [17, 18]. However, successful implementation faces substantial organizational challenges beyond technical performance. Healthcare managers must navigate trade-offs when deciding whether to develop local models, adopt external solutions, or adapt existing models to their institutions. These decisions are further complicated by differences in electronic health record systems, organizational structures, and availability of local training data [11, 19].

The development of generalizable predictive models in healthcare, particularly for ICUs, is an active area of research [20]. Many studies have focused on building and externally validating ML models, but results are often mixed when these models are applied in new clinical environments [21–23]. Advanced techniques like federated learning [24] or transfer learning [25] aim to improve model performance across sites, especially when local data is limited.

However, current research faces several limitations in addressing generalizability challenges. First, the impact of model complexity on generalizability remains largely underexplored, particularly in the context of clinical environments. Second, little attention has been given to the role of local training data. In particular, it is unclear whether small datasets can be used to optimize a locally developed model, or if it would be more effective to adopt a more sophisticated external model that has been trained on a much larger dataset. Third, most studies rely on aggregate performance metrics without examining the underlying reasons why models succeed or fail in new settings. As a result, healthcare managers lack clear guidance on when and how externally developed ML models can be safely applied to clinical and operational decision-making.

To address these critical gaps in knowledge, our study undertakes a systematic investigation into the factors shaping ML model generalizability in the ICU context. Our research is guided by three central research questions:

**RQ1:** How does model complexity influence cross-hospital generalizability of ICU prediction models?

**RQ2:** When local training data is limited, to what extent do external models or additional external data improve predictive performance?

**RQ3:** How can interpretability techniques reveal why models succeed or fail when transferred between healthcare institutions?

Through answering these questions, we aim to provide healthcare managers with guidance for identifying suitable models and data strategies when implementing predictive systems, particularly in settings with limited local data availability.

Our investigation explores the generalizability of ML models across two distinct ICU environments: a European University Hospital (EUH) dataset from Universitätsklinikum Carl Gustav Carus Dresden, Germany, and the publicly available Medical Information Mart for Intensive Care (MIMIC)-IV database. This cross-institutional approach allows us to assess how model complexity influences generalizability when ICU prediction models are transferred between healthcare institutions. We focus on three commonly studied ICU prediction tasks: mortality, length of stay, and readmssion to ensure clinical relevance and validity. In addition, we consider the impact of data availability. In particular, we simulate contexts where access to local data is limited. To deepen our understanding of these dynamics, we apply interpretability techniques to identify clinical features contributing to shifts in model behavior between settings. This approach provides practical insights into model selection, data requirements, and the risks associated with implementing externally developed models in new clinical environments.

Our analysis yields three key contributions for healthcare management. First, we demonstrate that model complexity is a key factor in cross-hospital generalizability, with low and medium complexity models significantly outperforming high complexity alternatives when transferred between institutions. Statistical analysis across three ICU prediction tasks reveals that more complex models experience disproportionate performance degradation when applied to different hospitals.

Second, we empirically identify patterns in data availability that can inform model selection strategies. Across prediction tasks, low complexity models perform well with a small amount of local training data, whereas medium and high complexity models require progressively larger datasets to achieve favorable local performance. This suggests that institutions with limited local data may benefit more from training low or medium complexity models locally than from adopting more complex external solutions.

Third, we demonstrate how interpretability techniques can reveal why models succeed or fail when transferred across hospitals, identifying specific clinical features where consistent physiological relationships enable successful deployment versus those where hospital-specific data collection practices create deployment risks. This provides healthcare managers with practical tools for validating external models without requiring access to proprietary training data.

The remainder of this paper is structured as follows. Section 2 reviews relevant literature on ICU prediction tasks, model complexity-generalizability relationships, and interpretability techniques. Section 3 details our experimental design, data sources, model selection, and evaluation approach for assessing cross-hospital generalizability. Section 4 presents our empirical findings across generalizability evaluation, data availability analysis, and interpretability insights. Section 5 discusses our key findings, their practical and theoretical implications for healthcare management, and identifies future research directions, while Section 6 concludes our work.

## Research background

Understanding the generalizability of ML models in healthcare requires examining three key aspects that inform our research approach. First, we establish the clinical context by examining the specific prediction tasks where ML models are deployed in ICU settings and their relevance to clinical decision-making (Section 2.1). Second, we discuss the aspect of local data availability and explore how ML model complexity may affect generalizability across hospitals (Section 2.2). Finally, we examine how different levels of model complexity relate to interpretability and how the latter can be used as a tool for a better understanding of generalizability patterns (Section 2.3). Together, these aspects provide the foundation for our empirical investigation of which modeling approaches are most suitable for cross-hospital deployment.

### ICU machine learning tasks and clinical decision making

ML has shown immense promise in healthcare, offering powerful tools to support clinical decision-making by creating rapid and accurate predictions based on complex, high-dimensional data [41–44]. In high-stakes environments such as the ICU, ML models are developed to predict patient outcomes, including the risk of life-threatening complications [45], readiness for transfer [39], length of stay [5], and likelihood of readmission [46]. These predictions can inform treatment decisions and help optimize resource allocation [36, 47, 48].

Our investigation focuses on three critical ICU prediction tasks that directly support clinical decision-making: *mortality*, *length of stay* in the ICU, and *readmission* to the ICU after discharge. These tasks were selected because they represent common applications of ML in intensive care settings and provide diverse profiles for evaluating model generalizability across different hospitals [12, 49].

Predicting a patient’s *mortality* is a common approach to assessing illness severity and acuity. As a definitive and consistently recorded outcome, mortality is frequently used as a target in ICU-focused ML research [5, 12]. Developed models are typically benchmarked against established scores such as APACHE IV [50] and SAPS III [51]. The goal is to identify high-risk patients early, enabling timely ICU transfers or staffing adjustments to ensure appropriate care. Studies have shown that patient mortality risk is closely linked to their acuity [26], and that patients with a high risk of dying often require more intensive nursing care [27]. As a result, acuity is commonly used in nurse-to-patient assignment problems to support balanced and fair workload distribution [28–30].

Estimating a patient’s *length of stay* is crucial for ICU resource planning and discharge coordination. Scheduling models, such as those by Turhan and Bilgen [34] and Heider et al. [35], rely on historical length of stay data to optimize patient admissions and surgical scheduling. Recent work by Shi et al. [36] leverages ML to handle long-tailed length of stay distributions, enhancing scheduling in complex settings. Individual length of stay predictions can further support early discharge planning by informing patients and caregivers about care plans and warning signs [37]. Moreover, prolonged ICU length of stay has been associated with increased resource use. Identifying patients at risk of prolonged stay can prompt a search for alternative care options, underlining the importance of accurate length of stay forecasting for both operational and clinical outcomes [52].

Predicting a patient’s *readmission* is essential for supporting safer discharge decisions from the ICU. Critical care professionals often base their discharge decisions on workload pressure and the ongoing demand for ICU beds [53]. In resource-limited settings, clinicians frequently face the challenge of identifying patients who are stable enough to be transferred to a general ward in order to free up ICU capacity for more critical cases. However, these decisions are complex, and readmissions to the ICU are associated with worse outcomes, including higher mortality rates, longer hospital stays, and increased costs [7, 54]. Identifying patients at risk of readmission can support safer discharge decisions and improve continuity of care [2, 39]. ML models are being explored as a promising approach to anticipate post-discharge deterioration. Accurate prediction could help prevent avoidable harm, optimize patient flow, and reduce the strain on critical care resources [46, 55, 56].

Table 1 summarizes the clinical importance and decision-making applications of these three prediction tasks. As illustrated, each task addresses distinct operational challenges: mortality prediction supports acuity-based resource allocation and workload planning, length of stay prediction enables proactive capacity management and surgical scheduling, and readmission prediction informs discharge safety protocols and care transitions. The diversity of these clinical applications, spanning from immediate bedside decisions to strategic resource planning, provides an effective foundation for evaluating the generalizability of ML models in various ICU settings and for different situations.

**Table 1:** Clinical importance and decision-making applications of ICU prediction tasks

Prediction Task	Clinical Importance	Associated Decision Tasks
Mortality	Proxy for illness severity and acuity [26]; correlates with nursing workload [27]	Acuity-based nurse assignment [28–30]; nurse re-scheduling after workload disruption [31]; admission control [32]
Length of stay	Enables discharge date estimation [33]; lower daily nursing workload for long stay patients [27]	Admission planning [2, 34]; surgery scheduling with downstream ICU capacity [35, 36]; early discharge planning [37]
Readmission	Identifies post-discharge risk within specified time frames; quality indicator for care transitions; increased mortality risk and higher cost for readmitted patients [38]	Patient discharge and transfer decisions [39]; ICU readmission as quality measure [40]

While these prediction tasks offer clinical value, their successful implementation across healthcare institutions faces substantial organizational challenges that extend beyond technical performance. Healthcare managers must navigate complex trade-offs when deciding whether to develop models locally, adopt external solutions, or adapt existing models to institutional contexts [19]. These decisions are complicated by heterogeneity in electronic health record systems, organizational structure, and data availability, all of which can limit model generalizability [11]. Understanding how model complexity shapes both generalizability and organizational acceptance is therefore critical for effective healthcare management decision-making [57].

### Model complexity and generalizability

The following section provides context for our first two research questions. First, we question how model complexity might impact generalizability across hospitals (RQ1). Second, we discuss under what conditions it might be preferable to train simpler models locally rather than adopt or adapt complex, externally developed models, particularly when local data availability is limited (RQ2).

In our context, generalizability refers to a model’s ability to maintain satisfactory performance when applied to distinct patient populations beyond the initial cohort used during development [16]. This quality is crucial when predictive models are created using external data and deployed in new environments, such as different hospitals [17]. The importance of generalizability becomes pressing when considering the potential risks of applying ML models in new settings, where variations in patient demographics and healthcare practices can lead to inaccurate and harmful predictions [18].

Generalizability provides insight into how robust a model is across different contexts and settings, revealing a dimension of performance measurement beyond the local setting where training data originates [16]. This is particularly relevant when multiple sites may benefit from a single model rather than training individual models for every location. Such model sharing is especially valuable when training data is scarce and large sample collection is unfeasible [15].

Complex models are capable of learning intricate and highly specific patterns within the training data, but these patterns are not necessarily transferable to other locations. Recent studies investigating ML model applicability across hospitals have yielded mixed results. The widely adopted Epic Sepsis model exemplifies these divergent outcomes. Wong et al. [21] critique the model’s performance when applied to a Michigan, U.S. hospital and raise concerns about sepsis care quality, while Cull et al. [45] affirm its effectiveness using data from Greenville, U.S. Similarly, a Hepatitis B prediction model successfully transferred between Nigerian hospitals but failed when applied to an Australian cohort [22]. These conflicting findings underscore the challenges associated with ML model generalizability in healthcare.

In many real-world settings, hospitals have only limited local data available, which constrains their ability to train complex models from scratch. Recent research offers potential solutions through federated learning, which allows hospitals to collaborate on model training without sharing data [24, 58], and transfer learning, which adapts models trained at one hospital for use at another using small amounts of local data [25, 59]. However, while these studies measure and report model performance across scenarios, they often fail to identify the exact reasons for performance differences or why model adjustments are necessary. Furthermore, these studies rarely consider whether training a simpler model directly on the limited local data might sometimes yield higher predictive performance than adapting a complex external model.

We examine how different model complexity levels influence both local performance and cross-hospital generalizability. We hypothesize that high model complexity may result in excellent performance at the hospital where training data originated but struggle to generalize effectively to other hospitals due to "local overfitting", where models learn hospital-specific patterns that are not applicable in other settings [20]. Additionally, we investigate how the level of local data availability interacts with model complexity to shape performance.

The challenge of estimating the generalizability of a model before transferring it, is compounded by the interpretability limitations of complex models. As models become more flexible and incorporate vast amounts of data and features, it becomes difficult for clinicians to understand the relationship between input features and model predictions [60–62]. When such complex models are transferred to new hospitals it is often unclear whether they will perform reliably or how they will behave in unfamiliar settings.

### Model complexity and interpretability

The generalizability challenges discussed in the previous section raise a critical question: how can we understand why models succeed or fail when transferred across hospitals? This question directly connects to our third research question (RQ3) about using interpretability techniques to explain generalizability patterns. Understanding the relationship between model complexity and interpretability is essential for addressing this challenge.

Achieving high predictive performance in healthcare often requires ML models with high flexibility to capture complex patterns in high-dimensional data [41]. Flexible model types, such as artificial neural networks and boosted decision trees, are well-suited for learning non-linear relationships and intricate feature interactions [63]. However, greater flexibility often comes at the cost of increased model complexity, making the model structure harder to interpret as it captures multi-level and sometimes opaque patterns across features [64].

**Fig. 1:**
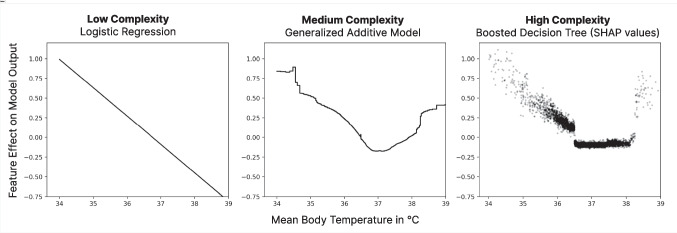
Low, medium, and high complexity models from left to right. An exemplary feature "Mean Body Temperature" has different effects on the model output for mortality prediction depending on the ML model complexity

This trade-off between flexibility and interpretability becomes particularly critical when considering cross-hospital generalizability. Interpretable ML refers to ML models and techniques that provide explanations for their predictions in terms understandable to human users, enabling them to comprehend, trust, and effectively manage the prediction process [65]. Without understanding the reasoning behind model predictions, it remains unclear why models succeed or fail in new environments or how they will respond to unfamiliar scenarios, making them potentially dangerous to use. When complex models fail to generalize effectively, it often remains difficult to identify the underlying reasons, since models may rely on hospital-specific patterns that do not transfer to other settings. Interpretable ML enables medical professionals to understand the decision logic behind ML models completely, ensuring that predictions align with domain knowledge and can be trusted for clinical decision-making across different environments [61, 66].

Two main strategies exist to address the interpretability challenge in the context of generalizability assessment. The first approach involves limiting model complexity through various constraints such as sparsity, linearity, or additivity [67]. By limiting complexity, the decision logic becomes more comprehensible to clinical stakeholders, making it easier to identify when and why models may struggle in new environments. Examples include logistic regression (linear and additive), decision trees (rule-based), and generalized additive models (additive but non-linear). However, this approach risks compromising the predictive performance that is critical in high-stakes healthcare applications [64].

The second strategy maintains model flexibility while applying additional techniques to reconstruct the decision logic of complex models with post-hoc explanation methods [67]. Common post-hoc explanation methods include SHapley Additive exPlanations (SHAP) [68] and Local Interpretable Model-agnostic Explanations (LIME) [69]. These methods provide insights into learned feature-outcome relationships, such as identifying positive or negative feature effects, determining feature importance, and revealing trends across different feature values. For generalizability assessment, these techniques can reveal which features contribute to successful or failed cross-hospital transfer. However, post-hoc explanations are approximations to the underlying model rather than exact representations [65].

Figure 1 demonstrates how the body temperature affects mortality prediction across three different model complexity levels. As model complexity increases from left to right, the interpretability of the feature’s effect becomes increasingly challenging to understand, illustrating the fundamental trade-off between model complexity and interpretability. This example highlights why interpretability becomes crucial when assessing whether learned patterns will generalize across different clinical environments.

While most research focuses on the trade-off between interpretability and performance when choosing between constraining complexity and using post-hoc explanations [62, 70], we additionally consider the complexity–generalizability trade-off. In this study, we focus on two key dimensions: evaluating models of varying complexity levels to understand the complexity-generalizability relationship, and applying interpretability techniques to explain why certain models generalize better than others across hospital settings. This comprehensive approach enables us to provide practical guidance for healthcare institutions considering cross-hospital ML model deployment.

## Methodology

Our methodology addresses the three research questions established in Section 1: how model complexity influences generalizability across hospitals (RQ1), to what extent external models or additional external data improve predictive performance when local training data is limited (RQ2), and how interpretability techniques can help understand these generalizability patterns (RQ3). This section outlines our research approach, data sources, model selection, experimental design, and evaluation approach to investigate these questions.

### Generalizability definition and operationalization

We define generalizability as a model’s ability to maintain satisfactory performance when applied to distinct patient populations beyond the initial cohort used during development [16]. In healthcare settings, this translates to how well models trained at one hospital perform when deployed at another institution with different patient demographics, clinical practices, and data collection procedures [11, 19].

We operationalize this concept using two datasets: $\mathcal {D}_s$ (source hospital) and $\mathcal {D}_t$ (target hospital), both containing the same *m* clinical features with $n_s = |\mathcal {D}_s|$ and $n_t = |\mathcal {D}_t|$ patients respectively. Each patient is represented by a tuple $(x_i, y_i)$, where $x_i \in \mathcal {X}^m$ describes the patient’s clinical features and $y_i \in \{0, 1\}$ indicates the task-specific outcome.

Our objective is to train ML models $f \in \mathcal {H}$ from a hypothesis space $\mathcal {H}$ that perform well on prediction tasks.

We systematically compare two scenarios: (1) *local models* where $f_t$ minimizes loss over patients in $\mathcal {D}_t$ and is evaluated in the same hospital, and (2) *external models* where $f_s$ minimizes loss over patients in $\mathcal {D}_s$ but is evaluated on $\mathcal {D}_t$. Comparing both approaches with models of different complexities provides insights into how model complexity impacts cross-hospital generalizability.

### Task definitions

We define three binary classification tasks using routinely collected ICU data. As detailed in Section 2.1 and summarized in Table 1, these tasks encompass diverse clinical decision-making contexts with different temporal prediction windows.

#### Mortality prediction

Indicating whether the patient died during the ICU stay. We use clinical data from the first 24 hours of ICU admission to enable early identification of high-risk patients.

#### Length of stay prediction

Separating long-stay patients ($\ge$7 days) from shorter stays, where length of stay is calculated as $(t^{\text {discharge}}_i - t^{\text {admission}}_i)/24$ hours. We use clinical data from the first 24 hours of admission to simulate an early prediction, which theoretically enables proactive capacity planning.

#### Readmission prediction

Indicating readmission within 72 hours of ICU discharge ($t^{\text {readmission}}_i - t^{\text {discharge}}_i \le 72$ hours). We use clinical data from the last 24 hours of the initial stay, including length of stay as an additional feature since it is available at discharge time. Patients who died during the initial ICU stay are excluded for this task to avoid prediction bias.

### Data sources and preprocessing

We use two large ICU datasets from geographically separate and independent healthcare institutions to evaluate cross-hospital generalizability. The datasets represent different healthcare systems and patient populations, providing an ideal testing environment for assessing model generalizability.

#### MIMIC-IV dataset

The Medical Information Mart for Intensive Care (MIMIC)-IV is a large-scale research dataset consolidating patient records from Beth Israel Deaconess Medical Center, collected between 2008 and 2022. MIMIC-IV is publicly available and represents one of the most widely used healthcare databases in ML research. For our study, we utilize 94458 de-identified health records across five ICUs within the medical center [71].

#### European university hospital (EUH) dataset

We utilize a proprietary dataset from Universitätsklinikum Carl Gustav Carus Dresden, Germany, covering surgical and anesthetic ICU admissions between 2004 and 2023. The dataset includes demographic and medical parameters comparable to MIMIC-IV data and encompasses 25062 patients.^1^

#### Feature selection and alignment

We selected 28 clinical features based on their prevalence in prior ICU models, availability in both datasets, and input from clinical experts. To support generalizability, we focused exclusively on routinely collected patient measurements such as vital signs and laboratory values that are typically available within the first few hours of ICU admission. We deliberately excluded variables directly related to clinical decisions or contextual information, such as admission reasons, medications, or treatment choices, as these are more likely to vary across hospitals and may reduce model comparability.

Both datasets undergo standardized preprocessing to ensure consistency: (1) unit alignment between datasets (e.g., converting glucose from mmol/L to mg/dL), (2) age adjustment in EUH data to match MIMIC-IV’s anonymization protocol (maximum age 90), (3) outlier removal using predefined clinical thresholds, (4) temporal aggregation using mean values over specified time windows, (5) exclusion of patients with >50% missing features, (6) standardized feature scaling and encoding, and (7) k-nearest neighbors imputation for remaining missing values.

Table 2 provides a comparative summary of the 28 clinical features for both datasets, showing mean values, standard deviations, and missing data rates. Notable differences include higher missing rates for certain features in EUH (e.g., respiratory rate: 41.9% vs. 0.23%) and MIMIC-IV (e.g., lactate: 39.98% vs. 0.27%), reflecting different data collection practices across institutions. Detailed explanations of all clinical features and specific outlier removal thresholds are provided in Appendix A.

**Table 2:** Comparison of MIMIC-IV and EUH data for 28 features. Mean values, standard deviations (std), and missing data rates are shown for each dataset. Note, that for the readmission task, we extracted the same features from the last 24h instead of the first 24h, resulting in slightly different values (see Appendix A)

Feature	MIMIC-IV			EUH		
	Mean	Std	Missing (%)	Mean	Std	Missing (%)
Age	63.67	16.41	0.00	64.04	16.62	0.01
Weight	82.65	23.75	2.49	77.74	19.61	37.60
Temperature	36.68	0.82	19.11	36.76	0.75	6.81
Respiratory Rate	19.26	3.82	0.23	17.22	3.97	41.91
Heart Rate	85.48	15.85	0.10	83.48	16.86	0.28
Glucose	136.73	48.38	0.87	138.23	35.51	0.28
Mean Blood Pressure	78.28	11.13	0.42	83.75	12.21	17.29
Potential Hydrogen	7.37	0.07	38.48	7.42	0.05	48.50
Glasgow Coma Scale Total	12.17	3.32	0.17	11.55	4.53	56.31
Gender (Female %)	43.27	–	0.00	40.24	–	0.00
Partial Pressure of O2	134.53	46.77	52.99	96.06	29.96	0.37
Fraction of Inspired O2	54.70	14.74	46.37	30.71	10.42	1.29
Potassium	4.18	0.55	0.16	4.13	0.42	0.38
Sodium	138.44	4.48	0.39	138.84	4.27	0.63
Leukocytes	12.21	7.75	1.06	11.49	5.92	4.89
Thrombocytes (Platelets)	205.75	106.81	0.61	223.60	109.84	4.82
Bilirubin	1.96	4.37	56.37	0.99	1.49	20.60
Bicarbonate	23.41	4.31	0.19	26.11	3.27	0.31
Hemoglobin	10.47	2.02	0.55	10.58	1.78	0.00
Prothrombin Time	1.45	0.61	15.06	1.37	0.35	5.15
Aspartate Aminotransferase	112.74	225.99	56.75	78.91	159.30	16.47
Alanine Aminotransferase	87.34	203.13	56.53	61.30	135.05	19.86
Partial Pressure of CO2	41.21	9.65	52.26	40.92	6.24	0.30
Albumin	3.16	0.60	73.22	2.98	0.54	28.64
Anion Gap	14.07	3.31	0.77	6.16	2.92	71.97
Lactate	2.16	1.53	39.98	1.48	1.57	0.27
Urea Nitrogen	26.32	21.66	0.23	41.42	32.20	15.33
Creatinine	1.43	1.50	0.10	1.07	0.99	5.79
Mortality (%)	7.30	–	0.00	5.70	–	0.00
Length of stay (%)	14.30	–	0.00	9.2	–	0.00
Readmission (%)	6.50	–	0.00	7.80	–	0.00

Despite these differences, a convex hull analysis (Appendix B) indicated substantial overlap in feature ranges (over 90%) between the two datasets, a pattern that generally suggests strong potential for cross-hospital generalizability [72].

### Model selection and complexity levels

We evaluate six ML models representing three distinct complexity levels, enabling systematic investigation of the relationship between model complexity and cross-hospital generalizability. As discussed in Section 2.3, model complexity affects both predictive performance and interpretability, with potential implications for generalizability that we investigate empirically.

#### Low complexity models

Logistic Regression (LR) serves as our baseline model, providing linear and additive relationships between features and outcomes with high interpretability. Decision Trees (DT) offer rule-based predictions through sequential splits that form clear decision paths. When constrained to shallow depths, DTs remain highly interpretable while capturing non-linear patterns through discrete splits.

#### Medium complexity models

Generalized Additive Models (GAMs) maintain the additive property of linear models while allowing non-linear feature-outcome relationships. We evaluate two GAM implementations: Explainable Boosting Machine (EBM) [73], which uses bagged and boosted tree ensembles to learn step functions, and Interpretable Generalized Additive Neural Networks (IGANN) [74], which employs extreme learning machines for gradient boosting. Both models provide inherent interpretability through visualizable feature shape functions while offering greater flexibility than linear models.

#### High complexity models

Extreme Gradient Boosting (XGB) and Multilayer Perceptrons (MLP) represent highly flexible models with universal approximation properties. XGB uses gradient boosting with decision trees to capture complex feature interactions and non-linear patterns. MLP employs multiple hidden layers with non-linear activation functions, enabling the learning of highly complex decision boundaries. Both models can approximate any continuous function on a compact domain but require post-hoc explanation methods, such as SHAP or LIME, for interpretability analysis.

All models are implemented using standard ML libraries with identical preprocessing pipelines. Implementation details are provided in Appendix C.

### Experimental design and scenarios

Our experimental design evaluates model generalizability across multiple dimensions in order to address our research questions. We implement three complementary approaches. First, we conduct a generalizability evaluation to investigate how model complexity influences cross-hospital generalizability (RQ1). Second, we perform a data availability analysis to examine how external models or additional external data affect predictive performance (RQ2). Third, we perform an interpretability analysis to explore why models succeed or fail (RQ3).

#### Generalizability evaluation

We evaluate model generalizability by comparing two primary training scenarios for each model and task combination: (1) *local data* trained and tested on the same hospital dataset, representing the standard scenario where sufficient local data is available, and (2) *external data* trained on one hospital dataset and tested on the other, representing the scenario where models must transfer across institutions. This bidirectional evaluation (EUH $\leftrightarrow$ MIMIC-IV) provides comprehensive assessment of generalizability in both directions, as transfer may not be symmetric due to dataset characteristics.

**Fig. 2:**
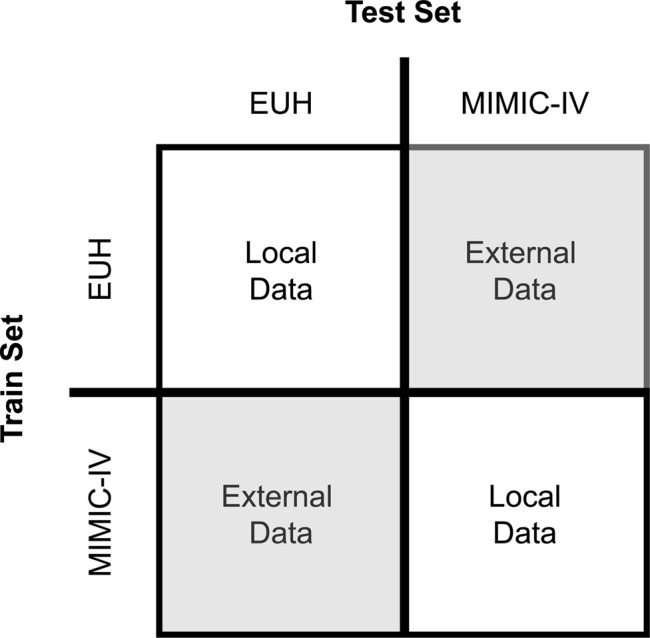
Different evaluation and training settings are indicated by color: white for models trained and tested on local data and grey for models trained on local data and tested on external data

Figure 2 illustrates these training and evaluation scenarios. Local scenarios (white) represent optimal conditions where models are trained and tested on data from the same institution. External scenarios (light gray) assess generalizability by testing models trained at one hospital on data from another hospital. This design directly addresses RQ1 by enabling systematic comparison of how different model complexity levels affect cross-hospital performance.

#### Data availability analysis

To simulate real-world constraints faced by institutions with limited local data, we conduct experiments with varying local data availability. We incrementally increase local training samples from 250 to 16000, focusing on the EUH dataset as the target institution. For each sample size, we compare two training approaches: (1) models trained exclusively on the limited local data, and (2) models trained on the limited local data supplemented with the complete external MIMIC-IV dataset. This analysis identifies "break-even points" where local-only models begin to outperform externally-supplemented models, answering RQ2, and providing practical guidance for institutions with limited data resources.

#### Interpretability analysis

To address RQ3 and understand the mechanisms underlying generalizability patterns, we apply interpretability techniques to examine feature-outcome relationships across datasets and models. For inherently interpretable models (LR, DT, GAMs), we visualize the direct model logic and feature effects. For complex models (XGB, MLP), we employ SHAP values [68] to provide post-hoc interpretability. SHAP values assign approximated contribution scores to individual feature values for each prediction, enabling generation of global explanation plots that show how specific features affect predictions across the entire dataset. This approach allows consistent interpretability analysis across all model types and identifies specific features where models learn consistent versus inconsistent relationships across hospitals, revealing potential sources of generalizability challenges.

**Fig. 3:**
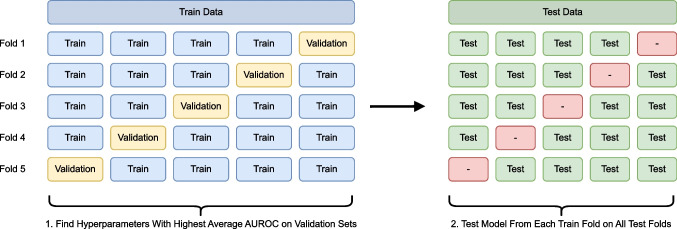
Overview of the training and evaluation strategy. Hyperparameters are optimized using 5-fold cross-validation on the training data, selected based on the average validation AUROC. Using these hyperparameters, five models are created on each training fold and individually evaluated on all five test folds, resulting in 25 evaluations per model and scenario

### Evaluation strategy and metrics

We developed an evaluation protocol designed to provide robust generalizability assessments.

#### Performance metrics

We use Area Under the Receiver Operating Characteristic Curve (AUROC) as our primary performance metric. AUROC is widely adopted for binary classification in ICU prediction tasks [12, 75] and offers threshold-independent evaluation, enabling fair comparison across hospitals without requiring model calibration. This metric is particularly suitable for our generalizability assessment because it remains consistent across different prevalence rates and decision thresholds. Thus, it is generally recommended for model comparisons [76].

#### Generalizability assessment

We measure generalizability using two complementary criteria that capture different aspects of cross-hospital performance.

*Model-specific Generalizability Loss* ($\Delta _{\text {spec}}$) measures the performance degradation experienced by each model when transferred between hospitals:

1 $$\begin{aligned} \Delta _{\text {spec}}(f_s) = \text {AUROC}_{D_t}(f_s) - \text {AUROC}_{D_s}(f_s), \end{aligned}$$

where $f_s$ is a model trained on source hospital $D_s$ and evaluated on target hospital $D_t$. This metric reflects the expected performance drop when externally trained models are applied locally.

Since a model with larger model-specific losses may be preferable in external scenarios due to its potentially higher base performance, we consider a second metric.

*Comparative Generalizability Loss* ($\Delta _{\text {comp}}$) measures performance relative to the best-transferring model:

2 $$\begin{aligned} \Delta _{\text {comp}}(f_s) = \text {AUROC}_{D_t}(f_s) - \max _{f_i \in \mathcal {F}_s} \text {AUROC}_{D_t}(f_i) , \end{aligned}$$

This metric highlights the performance gap between a given model and the optimal choice for cross-hospital deployment, providing practical guidance for model selection in external scenarios.

#### Evaluation protocol

We implement a standardized evaluation strategy to ensure reliable performance estimates and maintain strict train-test separation in all scenarios. Each dataset is initially split into training and test sets in a 80/20 ratio, and this division is maintained throughout all experiments.

Within the resulting train and test set, five slightly different folds are generated in a cross-validation pattern. This results in five training and five test folds for each hospital. For each experiment, we optimize the hyperparameters via grid search with cross-validation on the five train folds. We use ranges informed by established best practices (see Appendix C for details). The optimal parameters are then selected based on the average validation AUROC.

Using the optimal parameters found, we then train five models (one per training fold) and evaluate each on the five corresponding test folds. This results in 25 evaluations per model and scenario. This design yields robust performance estimates with uncertainty estimates, while strictly preventing any overlap between the train and test data, a critical consideration when the data come from different hospitals. Figure 3 visualizes our evaluation strategy.

#### Statistical analysis

The 25 evaluations per model enable robust statistical comparison across models and scenarios. Following Demšar [77], we employ the non-parametric Friedman test to detect significant differences in model performance (for both model-specific and comparative generalizability loss), followed by the Nemenyi post-hoc test for pairwise comparisons. These rank-based tests assign the lowest rank to models that lose the least performance when tested on external hospital datasets, providing a robust measure of generalizability. Therefore, lower ranks are better. This approach handles multiple comparisons effectively and allows visualization of critical differences between models [77]. Further details on the ranking procedure can be found in Appendix D.

## Results

This section presents our results studying the relationship between model complexity and cross-hospital generalizability in ICU outcome prediction. Following the methodology outlined in Section 3, we systematically evaluate six models of varying complexity across three prediction tasks using data from two geographically distinct healthcare institutions.

Section 4.1 reports model performance across local and external scenarios with statistical assessment of generalizability (RQ1). Section 4.2 examines how data availability constraints interact with model complexity to inform practical deployment decisions (RQ2). Section 4.3 applies interpretability techniques to reveal why certain models succeed or fail when transferred between institutions, providing deeper insights into the observed generalizability patterns (RQ3).

### Generalizability evaluation

We evaluate model generalizability by comparing local models (trained and tested on the same hospital dataset) with external models (trained on one hospital dataset and tested on another). Our analysis shown in Table 3 reveals consistent patterns across all three ICU prediction tasks, with local models systematically outperforming external models. However, the magnitude and characteristics of generalizability challenges vary substantially across prediction tasks.

**Table 3:**
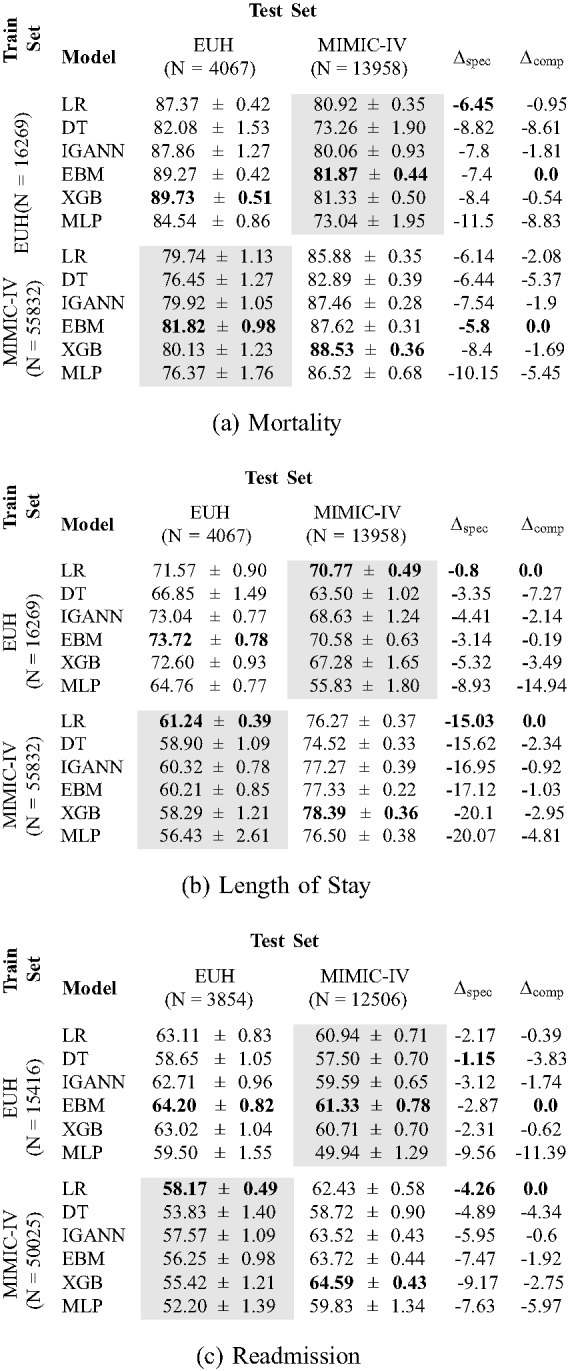
Model performance (AUROC ± standard deviation) for the three ICU classification tasks across train and test set combinations. Gray fields show scenarios with external test sets. $\Delta _{\text {spec}}$ and $\Delta _{\text {comp}}$ show model-specific and comparative generalizability losses

#### Mortality prediction

Mortality prediction demonstrates moderate and relatively symmetric generalizability across both transfer directions (Table 3a). Local models achieve strong performance on both datasets, with high complexity models slightly outperforming others (XGB: 89.73 on EUH, 88.53 on MIMIC-IV; EBM: 89.27 on EUH, 87.62 on MIMIC-IV).

The transfer shows relatively balanced patterns in both directions: models trained on EUH and tested on MIMIC-IV experience model-specific generalizability loss ($\Delta _{\text {spec}}$) ranging from 6.45 (LR) to 11.5 (MLP) AUROC points, while models trained on MIMIC-IV and tested on EUH show similar ranges from 6.14 (LR) to 10.15 (MLP) points. EBM demonstrates the best external performance when transferred in both directions, achieving comparative generalizability loss ($\Delta _{\text {comp}}$) of 0.0, indicating it is the best-performing external model for this task.

#### Length of stay prediction

Length of stay prediction exhibits the most pronounced and asymmetric generalizability challenges among all three tasks (Table 3b). Local models perform well (EBM best on EUH: 73.72; XGB best on MIMIC-IV: 78.39), but external performance varies highly by transfer direction.

The transfer shows highly asymmetric patterns: models trained on MIMIC-IV and tested on EUH experience severe model-specific generalizability loss ($\Delta _{\text {spec}}$) ranging from 15.03 (LR) to 20.1 (XGB) points, while models trained on EUH and tested on MIMIC-IV show much smaller drops from 0.8 (LR) to 8.93 (MLP) points. LR achieves the best external performance in both directions, outperforming more complex alternatives despite its simpler architecture. Interestingly, models trained on EUH data transfer reasonably well to MIMIC-IV, whereas models trained on MIMIC-IV show significant performance losses on EUH. This asymmetric pattern in generalizability suggests a fundamental difference in how length of stay is determined between hospitals.

#### Readmission prediction

Readmission prediction demonstrates the most stable generalizability characteristics, with moderate performance drops and less pronounced directional asymmetry (Table 3c). Local models show modest performance differences (EBM best on EUH: 64.20; XGB best on MIMIC-IV: 64.59), with overall performance levels being more modest than mortality prediction.

The transfer patterns are relatively balanced: models trained on EUH and tested on MIMIC-IV experience model-specific generalizability loss ($\Delta _{\text {spec}}$) ranging from 1.15 (DT) to 9.56 (MLP) points, while the reverse direction shows similar ranges from 4.26 (LR) to 9.17 (XGB) points. EBM and LR achieve best external performance ($\Delta _{\text {comp}}$ = 0.0) in different transfer directions, indicating consistent generalizability for medium and low complexity models.

**Fig. 4:**
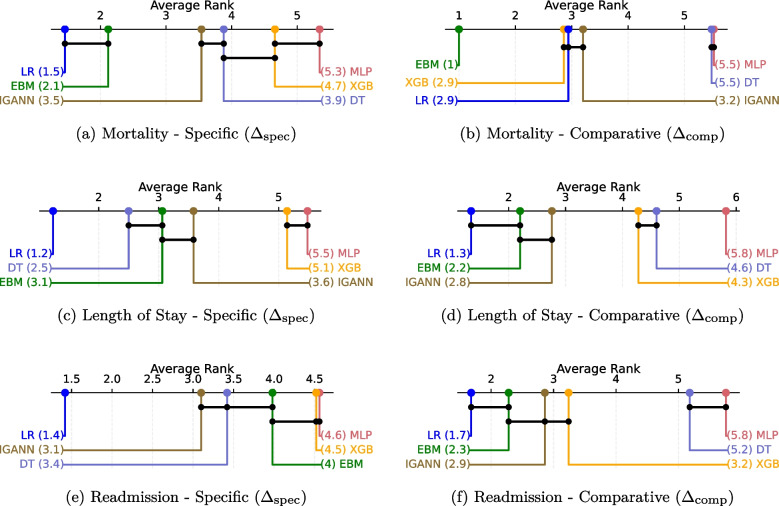
Critical difference diagrams for generalizability assessment. Lower average ranks indicate better performance of externally trained model. Left column: Model-specific generalizability loss ($\Delta _{\text {spec}}$). Right column: Comparative generalizability loss ($\Delta _{\text {comp}}$). Non-significant differences are connected with horizontal lines ($^*p> 0.05$)

#### Cross-task generalizability patterns

Three consistent patterns emerge across all prediction tasks. First, high complexity models (XGB, MLP) achieve competitive local performance but suffer disproportionate degradation when transferred, with MLP consistently showing the largest model-specific generalizability loss across all tasks (7.63-20.07 points). Second, medium complexity models, particularly EBM, empirically provide a better balance between local performance and cross-hospital generalizability. EBM performs competitively with XGB across all tasks (typically within 1 AUROC point) while demonstrating superior generalizability. Third, low complexity models like LR achieve more modest local performance levels, but demonstrate remarkable transfer stability with minimal performance drops.

The variation in generalizability across tasks highlights the importance of task-specific considerations in model deployment decisions. Mortality prediction offers relatively stable transfer characteristics, length of stay prediction requires careful assessment of hospital-specific practices, while readmission prediction emerges as the most consistently generalizable task across both hospitals.

#### Statistical assessment of generalizability

Figure 4 presents the statistical assessment of generalizability through critical difference diagrams, where models connected by horizontal lines show no significant differences ($p> 0.05$). This rank-based analysis, shown separately for the model-specific and comparative generalizability losses, reveals a consistent hierarchy: low and medium complexity models significantly outperform high complexity models in cross-hospital generalizability, extending the performance results in Table 3.

Model-specific generalizability loss ($\Delta _{\text {spec}}$, left column), measuring degradation relative to local performance, highlights LR as the most stable model across all tasks (ranks 1.5, 1.2, 1.4), significantly outperforming nearly all others. This supports the observation that simpler models generalize more consistently. Medium complexity models, especially EBM, show strong but task-dependent generalizability (ranks 2.1, 3.1, 4.0), with EBM and IGANN often indistinguishable ($p> 0.05$). By contrast, high complexity models generalize poorly, with MLP ranking worst (5.3, 5.5, 4.6) and XGB showing similar degradation. These results confirm that high complexity models suffer disproportionate loss when transferred.

**Fig. 5:**
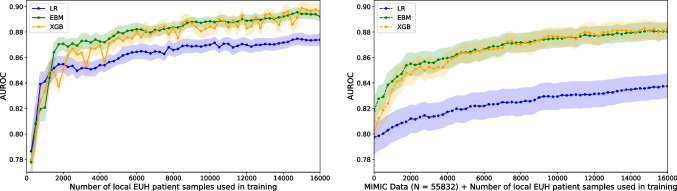
Model performance (AUROC) for mortality prediction over EUH sample size for LR, EBM, and XGB. Left: Models trained on EUH data only. Right: Models trained on same EUH samples supplemented with full MIMIC-IV dataset (additional 55832 samples). Shaded areas represent standard deviation

Comparative generalizability loss ($\Delta _{\text {comp}}$, right column), which directly compares external performance, refines these patterns. For mortality, EBM is optimal (rank 1.0), significantly outperforming all other models when transferred. For length of stay, where generalizability asymmetries are most pronounced, LR ranks best (1.3) but does not differ significantly from EBM (2.2). XGB (4.3) shows instability despite strong local performance. Readmission reveals broader equivalence among simpler models, with LR (1.7) and EBM (2.3) forming the top cluster, while MLP again ranks worst (5.8). The corresponding average rankings are provided in tabular format in Appendix D.

Overall, these assessments confirm that low- and medium-complexity models generalize better than high complexity models. Only LR and EBM achieve best external performance ($\Delta _{\text {comp}} = 0.0$) across all tasks and directions. High complexity models like XGB and MLP, despite competitive local AUROC, never emerge as best external models and degrade by 5–10 AUROC points more than simpler alternatives. Medium complexity models, particularly EBM, balance local strength with robust generalizability, while LR excels in both metrics, making it optimal for cross-hospital deployment. This establishes a clear trade-off: model complexity inversely correlates with cross-hospital generalizability (RQ1).

### Data availability analysis

To answer RQ2, and to simulate real-world data constraints, we incrementally varied local training samples from 250 to 16000 in steps of 250, using EUH as the target institution. For each sample size, we compared two training approaches: (1) models trained exclusively on the limited local data, and (2) models trained on the limited local data supplemented with the complete external MIMIC-IV dataset (55832 samples). We focus on three representative models (LR, EBM, XGB) that represent different complexity levels and previously demonstrated strong performance. We report results for mortality prediction, but similar trends are observed for the remaining prediction tasks on length of stay and readmission (see Appendix E for details).

#### Performance with varying local data availability

Figure 5 shows model performance across the full range of sample sizes. In the local-only scenario (left panel), all models demonstrate characteristic learning curves with rapid initial improvement followed by diminishing returns. LR reaches a performance plateau early, while EBM shows consistent improvement and XGB requires larger datasets for stable performance.

When increasing local data is supplemented with the complete MIMIC-IV train dataset (right-hand panel), the baseline performance of all models is substantially higher. Adding local EUH data improves the performance of all models. XGB and EBM benefit most from this. Furthermore, XGB shows greater stability and less variability when supplemented with external data. In contrast, LR shows the least improvement with additional local data.

#### Break-even point identification

**Fig. 6:**
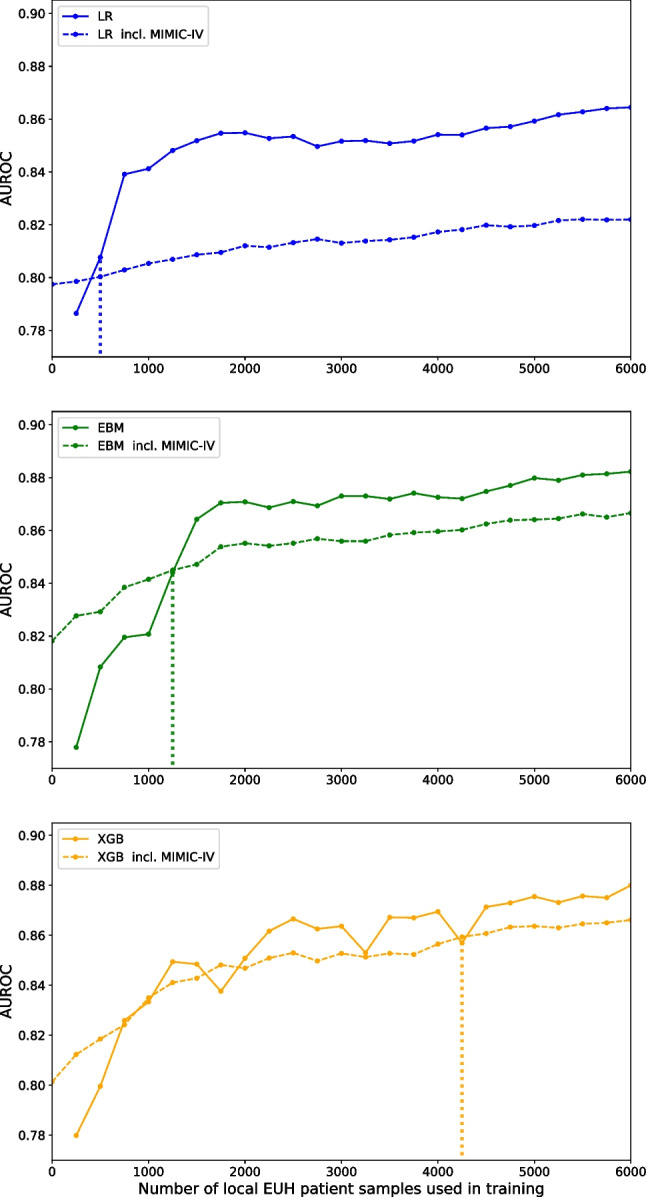
Break-even points for LR, EBM, and XGB models. Solid lines represent models trained on EUH data only; dashed lines represent models trained on EUH data supplemented with MIMIC-IV data. Vertical lines indicate break-even points identified through systematic sampling from 250 to 6000 EUH samples

We identify break-even points where local-only models begin to consistently outperform models supplemented with external data (Figure 6). These thresholds reveal a clear relationship between model complexity and data requirements: LR reaches break-even at approximately 500 EUH samples, EBM at 1250 samples, and XGB at 4250 local samples to render external data unnecessary.

The systematic progression from 250 to 6000 samples allows identification of these transition points, where the solid lines (local-only) cross above the dashed lines (local + external). Beyond these break-even points, local-only models maintain consistent superiority, validating our generalizability findings from Section 4.1.

As mentioned, the results for length of stay and readmission prediction are very similar to those for mortality (see Appendix E). Taken together, these consistent findings across all tasks highlight an important trend: as model complexity increases, so does the amount of local data needed for a purely local model to surpass one using additional external data.

These findings relating to the three tasks provide healthcare institutions with guidance based on available data volumes. With smaller datasets (250–2000), low complexity models such as LR tend to perform best. With moderate data sizes (500–3000), medium complexity models such as EBM can offer advantages. Only with sufficiently large datasets (>4500) high complexity models like XGB become really competitive. Only when institutions have no local data or very small amounts, external models or datasets represent the best alternative. These approximate thresholds support data-driven ML adoption decisions and help institutions avoid over-investing in complex solutions that may underperform simpler local alternatives.

### Interpretability analysis

**Fig. 7:**
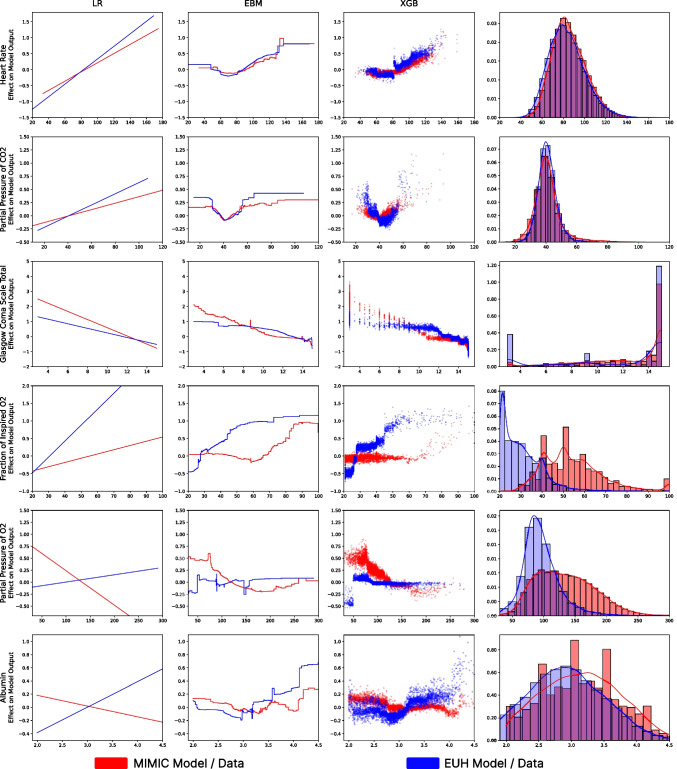
Comparison of feature-mortality relationships learned by LR, EBM, and XGB on EUH (blue) and MIMIC-IV (red) datasets. Histograms show feature distributions in each dataset, with density shown on the y-axis. For LR and EBM, plots directly reveal model decision logic. For XGB, SHAP values for EUH test data are shown

To understand the mechanisms underlying the generalizability patterns observed in Sections 4.1 and 4.2, we analyze the feature-outcome relationships learned by different models using interpretability techniques. This analysis addresses RQ3 by examining how these techniques can reveal why models succeed or fail when transferred between institutions.

Figure 7 illustrates these relationships for six key features across three models for mortality predictions. The figure compares models trained on EUH data (blue) with those trained on MIMIC-IV data (red), alongside histograms showing the feature distributions in each dataset. In the following, we focus on mortality, noting that results for length of stay and readmission are comparable (detailed visualizations for these tasks are provided in Appendix E). For LR and EBM, the plots directly reveal the models’ decision logic, whereas for XGB, we use SHAP values computed for individual patients in the EUH test set.

#### Consistent feature-outcome relationships

Analysis of key clinical features reveals varying degrees of consistency in learned relationships across the EUH and MIMIC-IV datasets (Fig. 7). Several features demonstrate robust, transferable relationships that explain strong generalizability performance for certain models.

*Heart Rate* shows remarkably consistent relationships across all models and both datasets. LR demonstrates similar linear trends, while EBM captures consistent non-linear patterns with minimum mortality risk at 70-80 bpm and increased risk at extremes. This consistency aligns with established mortality scoring systems used in intensive care, such as APACHE-IV and SAPS III [50], and suggests that heart rate contributes positively to model generalizability across hospitals.

*Partial Pressure of CO2* exhibits similar consistency, with all models learning comparable relationships across datasets. EBM reveals optimal values around 40 mmHg in both hospitals, with deviations in either direction associated with increased mortality risk. This pattern aligns with the established clinical reference range for partial pressure of CO2, which is typically between 32 and 46 mmHg [78]. These consistent patterns suggest that fundamental physiological relationships remain stable across different hospital settings.

*Glasgow Coma Scale* shows weaker predictive relationships in EUH compared to MIMIC-IV, likely due to differences in measurement frequency and recording practices for sedated patients [79]. The EUH dataset exhibits higher frequency of minimum scores (3), potentially reflecting different protocols for sedated patients, which weakens the feature’s discriminative power. However, the general association between lower scores and higher mortality risk remains consistent across both datasets.

#### Problematic feature variations

In contrast, several features show substantial differences in learned relationships across hospitals, revealing specific sources of generalizability challenges and explaining why models fail when transferred.

*Fraction of Inspired O2* demonstrates huge differences between datasets, both in distribution and learned relationships. The EUH dataset shows substantially lower mean values, likely due to different recording practices for non-ventilated patients, where room air is recorded as 21% oxygen concentration. This leads to opposing learned relationships: MIMIC-IV models associate lower fraction of inspired O2 with increased mortality risk, while EUH models show the reverse pattern. Such divergent relationships directly explain poor generalizability for models relying on this feature.

*Partial Pressure of O2* shows clear distributional differences between datasets, which may reflect distinct measurement procedures. In MIMIC-IV, the models identify meaningful relationships: LR shows a negative trend, whereas EBM and XGB capture a U-shaped curve linking very small values to higher mortality. By contrast, the EUH data exhibit much weaker associations with mortality, suggesting that partial pressure of O2 may be a less reliable mortality predictor in this hospital.

*Albumin* presents another problematic case where similar distributions mask opposing learned relationships. MIMIC-IV models correctly learn that higher albumin levels associate with lower mortality (consistent with medical literature [80]), while EUH models learn the opposite trend. This discrepancy suggests dataset-specific confounding factors that compromise model generalizability despite apparently similar data characteristics.

#### Model complexity and feature contributions

Differences in feature distributions across hospitals help to explain some of the challenges that models face when generalizing, as they affect how reliably predictive relationships can be established. However, we also identify features with very similar distributions that result in inconsistent feature-outcome relationships. Nevertheless, interpretability analysis shows that the overall trends in feature–outcome relationships are consistent across model types: LR, EBM and XGB all capture similar directions of effect, even for problematic features. This suggests that the disproportionately greater loss of performance of complex models cannot be attributed solely to these visible feature contributions. Rather, it suggests that complex models also exploit subtle, dataset-specific patterns that fail to transfer across hospitals. While problematic features may contribute to generalizability challenges, they do not fully explain the complexity–generalizability relationships observed.

However, interpretability techniques provide valuable insights, revealing both robust physiological relationships that support generalizability and problematic feature variations that undermine generalizability. In doing so, they directly address RQ3 and offer healthcare managers concrete methods for validating external models before deployment. This enables them to proactively identify features that may compromise performance in their specific hospital, potentially necessitating changes in data collection.^2^

## Discussion

The generalizability of ML models is a critical issue in healthcare analytics [20, 25]. Our study shows that ICU outcome prediction models can be transferred between hospitals, but with substantial differences depending on model complexity.

Across three prediction tasks, we conducted a systematic series of experiments with six models spanning different complexity levels, from LR with low complexity to GAMs with medium complexity and high complexity approaches like XGB and MLP. This design enabled us to assess the generalizability of each model type when transferred across hospitals, also for different simulated data availability settings.

Addressing our research questions, we find that model complexity is inversely related to cross-hospital generalizability, with low and medium complexity models proving more robust (RQ1). External models are not guaranteed to improve performance when local data is scarce and benefits depend on data availability, making locally trained simpler models often preferable (RQ2). Interpretability techniques provide essential tools for understanding generalizability problems, revealing both consistent physiological relationships and dataset-specific artifacts that compromise generalizability but are not fully able to explain the weaker generalizability of more complex models (RQ3).

These findings translate into actionable guidance for healthcare ML adoption decisions. Rather than defaulting to the most advanced available algorithms, healthcare managers should evaluate model complexity against their institutional data capacity and deployment objectives.

### Practical implications

These complexity-generalizability principles translate directly into actionable guidance for healthcare administrators and clinical decision-makers. The practical implications of our study underscore the potential advantages of employing low and medium complexity models when working with models trained on external data. While these models may have slightly lower performance on large local datasets, their improved generalizability makes them a reasonable choice in scenarios where local data is limited or only external data is accessible.

For institutions with limited data resources, our results suggest that simple locally trained models often outperform high complexity external models, regardless of the latter’s reported performance in other hospitals. With smaller datasets, low complexity approaches such as LR tend to perform best; with moderate data sizes, medium complexity models like EBM provide additional benefits; and only with sufficiently large datasets can high complexity models such as XGB become truly superior. External models or external data are only a viable option when local data are unavailable or extremely limited, or in rare cases where the clinical task demands the use of a highly complex model. This challenges current procurement practices that prioritize advertised performance over demonstrated cross-hospital generalizability and interpretability. Further, it supports calls for institution-specific modeling strategies over universal solutions [19, 20].

Our interpretability analysis provides a systematic approach for risk assessment when deploying external models (Section 4.3). By examining feature-outcome relationships, healthcare managers can identify potential deployment risks before implementation [66]. Features showing consistent relationships across hospitals (such as heart rate and partial pressure of CO2) provide reliable foundations for cross-hospital deployment, while those with divergent patterns (like fraction of inspired O2 and albumin) require careful validation and potentially customized approaches. This type of interpretive analysis can be conducted even in smaller hospitals without access to the training data of external models, making it applicable in scenarios where data sharing is limited. This addresses the critical need for validating external models to avoid poor allocation of ML resources that can lead to care inefficiency and suboptimal patient outcomes [81].

For resource allocation scenarios, our results demonstrate measurable impacts on operational decisions. To illustrate the practical relevance of our findings, we conducted a numerical study based on the nurse assignment problem, detailed in Appendix G, which relies on patient acuity estimates derived from mortality risk predictions. The analysis shows that using less generalizable external models can result in suboptimal clinical decisions. Even when the difference in predictive performance appears modest, the resulting misalignment in nurse workloads can be substantial. These findings emphasize the importance of generalizability in clinical ML and demonstrate the practical impact that prediction quality can have on healthcare operations and management.

In light of these findings, we strongly advocate the inclusion of interpretability analyses as a mandatory component of model validation when applying externally created models to new hospitals. This practice would not only increase the safety and reliability of ML applications in healthcare, but also foster greater trust and acceptance among healthcare professionals [61, 82]. By bridging the gap between model performance and clinical interpretability, we can better realize the potential of ML to improve patient care while preserving the critical role of human expertise in medical decision-making.

### Theoretical implications

Beyond these immediate practical applications, our findings have broader theoretical implications for healthcare ML research and emerging collaborative approaches.

Our study contributes to the growing body of evidence challenging the performance-interpretability trade-off in healthcare ML [65, 70]. The consistent superior cross-hospital generalizability of low and medium complexity models across diverse clinical tasks suggests that the healthcare domain may inherently favor interpretable, structured approaches over highly flexible, more complex architectures. This finding is particularly relevant because trust emerges as a critical factor in healthcare ML adoption, and complex algorithms create additional barriers for clinicians to trust ML-based technology [57, 62, 82]. Our results suggest that pursuing interpretable models may simultaneously improve both generalizability and organizational acceptance.

Our findings have important implications for emerging approaches like federated learning and transfer learning in healthcare [24, 25]. While these techniques attempt to leverage external data while preserving data privacy, our results suggest that simpler models trained only on local data often outperform complex externally trained approaches. This challenges the assumption that more data necessarily improves model performance and suggests that data quality and relevance may be more important than data quantity. The mixed results with the Epic Sepsis model across different datasets [21, 45] exemplify these generalizability challenges and highlight the need for systematic approaches to identify why models succeed or fail across institutions.

The interpretability insights reveal that successful cross-hospital deployment requires understanding local clinical practices and data collection protocols. While models of different complexity levels generally learn similar relationships between features and outcomes, data collection procedures substantially impact model performance and learned feature-outcome relationships. This finding supports arguments for standardizing clinical data collection and documentation across healthcare institutions [19], which could improve model generalizability.

The asymmetric generalizability patterns we observed, particularly for length of stay prediction, highlight the importance of understanding task-specific factors in model deployment. Unlike mortality, which reflects fundamental physiological relationships, outcomes influenced by institutional policies and clinical judgment (such as length of stay) might be inherently less generalizable across hospitals. Healthcare managers should consider these task characteristics when assessing ML applications and setting realistic expectations for cross-hospital model performance.

### Limitations and future research directions

While our study establishes clear principles for cross-hospital ML deployment, several limitations point toward important directions for advancing both healthcare management practice and research.

Our analysis focuses on two datasets representing European and North American healthcare systems, which may limit applicability to other geographic regions or healthcare delivery models. The complexity-generalizability relationships we identified should be validated across diverse healthcare systems, particularly those in resource-constrained settings where our findings about low complexity model advantages may prove most relevant for ML adoption decisions. This expansion could include different types of hospitals (e.g., rural vs. urban) and various geographical regions to capture a wider range of healthcare practices and patient populations.

While mortality, length of stay, and readmission represent common and clinically important ICU applications, other healthcare prediction tasks may exhibit different generalizability characteristics. Unlike mortality, which is well-documented and consistently labeled, other predictive targets such as sepsis onset or readiness for discharge have unique labeling processes that can present additional challenges [10]. These targets often involve more subjective assessments and may vary between institutions, potentially impacting model generalizability. Future research should investigate whether our complexity-generalizability principles extend to other clinical domains such as emergency medicine, surgical outcomes, or chronic disease management to improve our understanding of generalizability in less clear-cut scenarios.

Further, to ensure comparability, we restricted our analysis to models trained on structured tabular data. In real world practice, however, physicians integrate information from many sources, including imaging, clinical reports, laboratory values, and bedside monitoring, into an evolving assessment of the patient. Each new test or measurement can update the clinical picture and alter treatment decisions, underscoring the complexity of intensive care decision-making. While methods exist for individual data types such as imaging [83], clinical notes [84], or time series [85], a truly multimodal model that combines all of them with temporal dynamics has yet to be realized. Such models would require complex architectures, careful adaptation to local settings, and face major challenges in cross-hospital generalizability. Nevertheless, investigating their potential and the generalizability remains an exciting direction for future research. Our work provides only a first impression in this regard.

Our models relied on data-driven learning without explicit incorporation of established medical knowledge, despite our interpretability analysis revealing alignment with clinical understanding. However, our detailed interpretability analysis provides a strong foundation for this line of future research. By identifying specific features with inconsistent effects across datasets, such as fraction of inspired O2 and albumin (Section 4.3), our work offers a starting point for knowledge-guided approaches [86]. Future research should investigate knowledge-guided modeling approaches that incorporate established physiological relationships and clinical rules during model development, for instance by using established clinical rules to manage features with high variability or by incorporating known physiological relationships into the model’s learning process [87]. Such approaches could potentially address the generalizability gaps we observed by ensuring consistency with medical understanding across different institutional settings.

Finally, while our study addresses model interpretability from an analytical perspective, further exploration of how healthcare professionals interact with and trust these models in real-world scenarios could offer valuable insights. This could involve qualitative research methods to understand how clinicians interpret and utilize model predictions in their decision-making processes [10, 61, 62]. Understanding clinicians' decision-making processes, acceptance criteria, and workflow integration preferences could inform model selection and deployment strategies that better align with existing healthcare operations and foster greater trust and acceptance among healthcare professionals.

## Conclusion

Our study examined the cross-hospital generalizability of ML models for ICU outcome prediction using data from a European university hospital and MIMIC-IV. Across mortality, length of stay, and readmission tasks, we found that model complexity strongly shapes generalizability: simpler models (LR, GAMs) transfer more reliably than complex ones (XGB, MLP), which often overfit to local patterns. Interpretability analyses showed that while feature–outcome relationships are broadly consistent across models, variations in data collection across hospitals can create problematic features that limit generalizability. In practice, low complexity local models are best in low data settings, medium complexity models offer a strong balance of performance, interpretability, and generalizability, while high complexity models require large local datasets to be effective. External models or data are useful only when local data is scarce or unavailable. Overall, prioritizing simpler and interpretable models and data quality over algorithmic complexity provides safer, more generalizable insights for ICU outcome predictions. Complex models are only suitable as individual solutions for institutions with substantial local training data.

## Figures and Tables

**Fig. 8:**
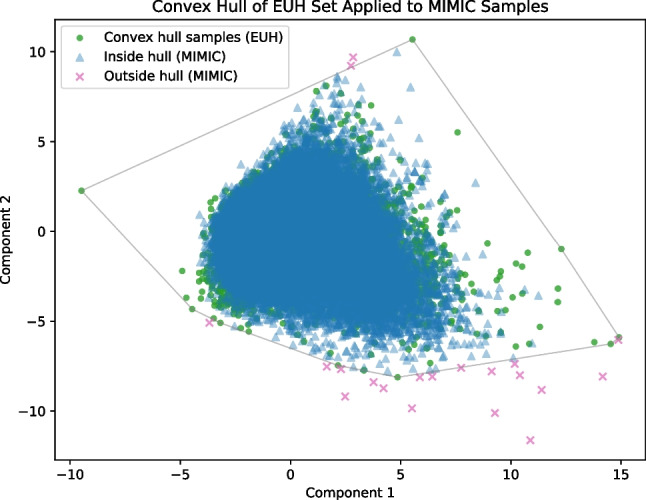
Convex hull for the EUH data using the full feature set. The analysis uses and shows the first two components from a PCA using only the training statistics from EUH to impute, encode, scale and decompose EUH (training / constructing convex hull) and MIMIC (test) into two components. MIMIC samples inside the hull of EUH data are marked with triangles and outside the hull marked with crosses

**Fig. 9:**
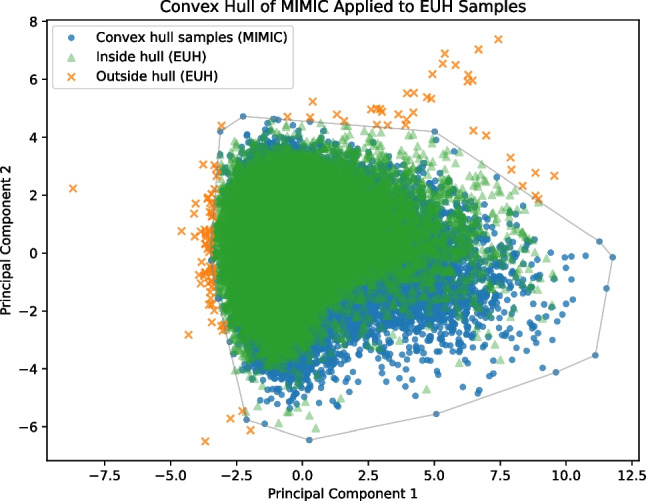
Convex hull for the MIMIC data using the full feature set. The analysis uses and shows the first two components from a PCA using only the training statistics from MIMIC to impute, encode, scale and decompose MIMIC (training / constructing convex hull) and EUH (test) into two components. EUH samples inside the hull of MIMIC data are marked with triangles and outside the hull marked with crosses

**Fig. 10:**
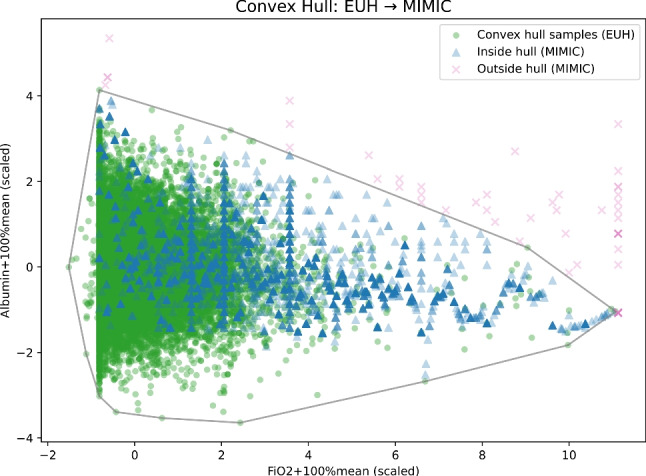
Convex hull for the EUH data using two features only: Fraction of inspired O2 (FiO2) and albumin. MIMIC samples inside the hull of EUH data are marked with triangles and outside the hull marked with crosses

**Fig. 11:**
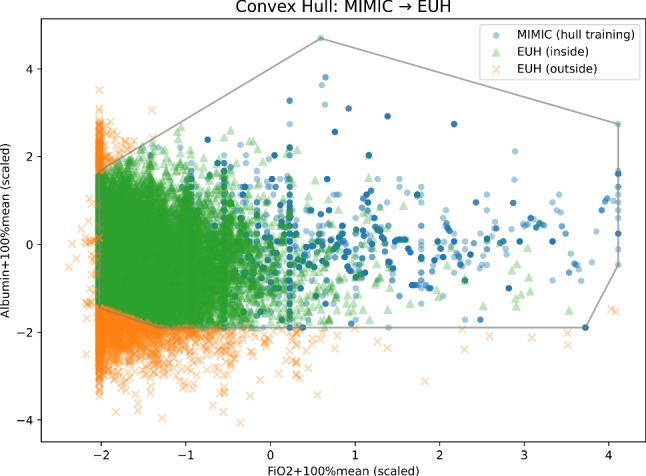
Convex hull for the MIMIC data using two features only: Fraction of inspired O2 (FiO2) and albumin. EUH samples inside the hull of MIMIC are marked with triangles and outside the hull marked with crosses

**Fig. 12:**
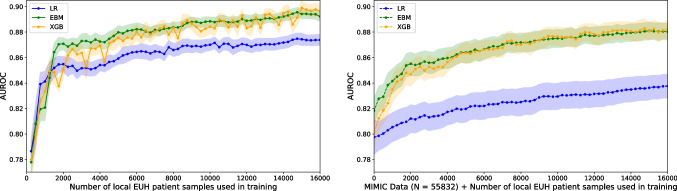
Model performance (AUROC) for mortality prediction over EUH sample size for LR, EBM, and XGB. Left: Models trained on EUH data only. Right: Models trained on same EUH samples supplemented with full MIMIC-IV dataset (additional 55832 samples). Shaded areas represent standard deviation

**Fig. 13:**
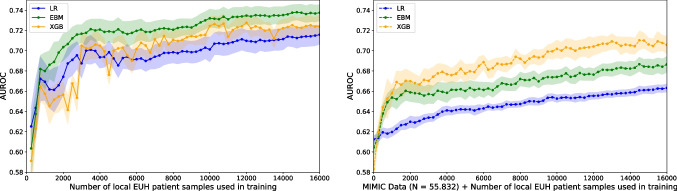
Model performance (AUROC) for length of stay prediction over EUH sample size for LR, EBM, and XGB. Left: Models trained on EUH data only. Right: Models trained on same EUH samples supplemented with full MIMIC-IV dataset (additional 55832 samples). Shaded areas represent standard deviation

**Fig. 14:**
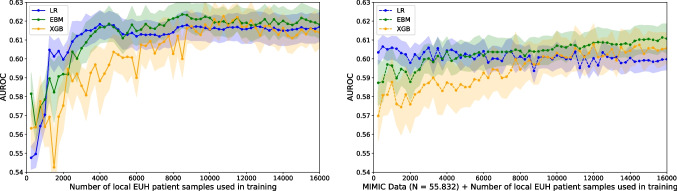
Model performance (AUROC) for readmission prediction over EUH sample size for LR, EBM, and XGB. Left: Models trained on EUH data only. Right: Models trained on same EUH samples supplemented with full MIMIC-IV dataset (additional 55832 samples). Shaded areas represent standard deviation

**Fig. 15:**
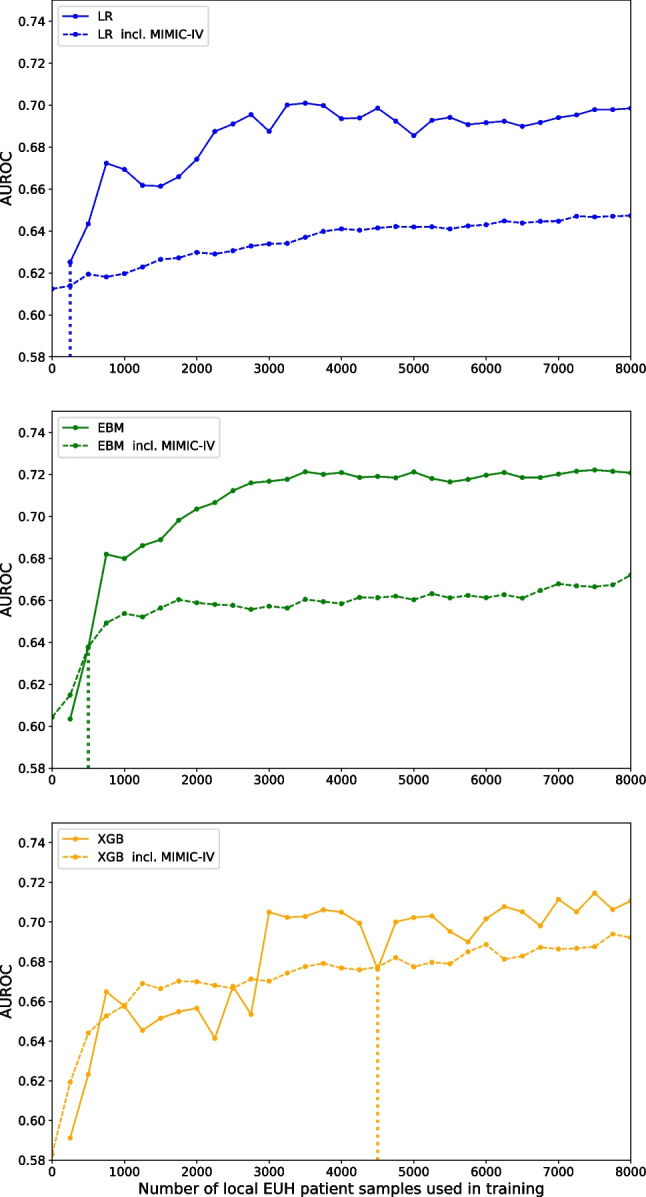
Break-even points for LR, EBM, and XGB models. Solid lines represent models trained on EUH data only; dashed lines represent models trained on EUH data supplemented with MIMIC-IV data. The x-axis shows the number of EUH samples used, while the y-axis displays AUROC values for length of stay predictions. Vertical lines indicate the break-even points

**Fig. 16:**
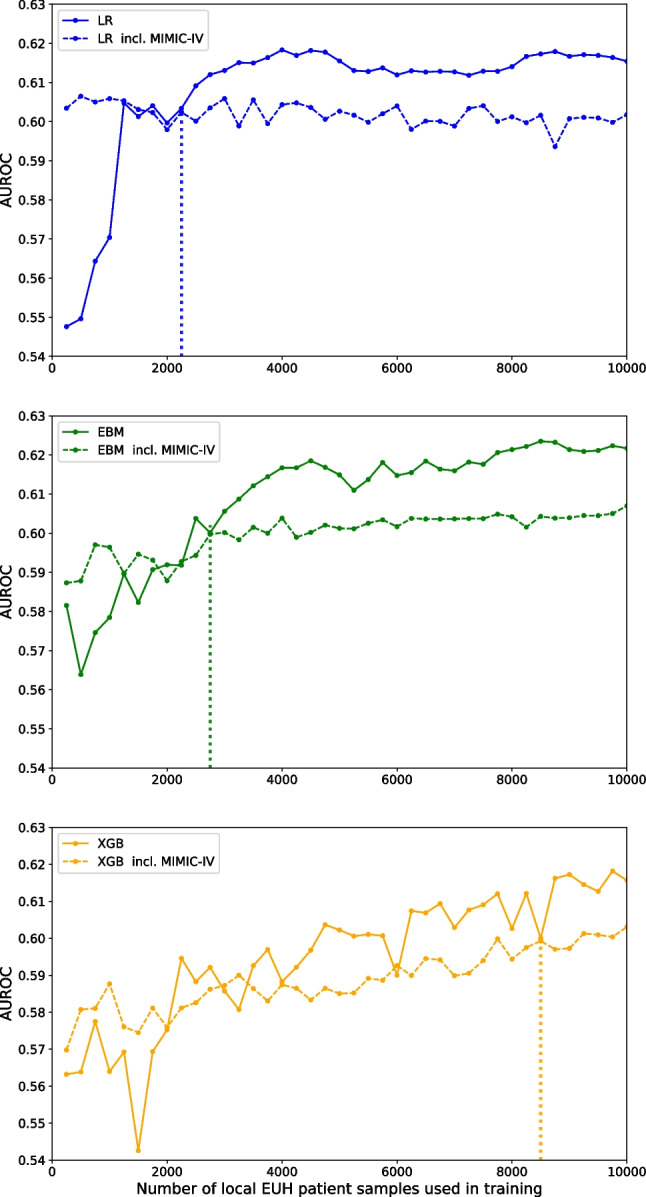
Break-even points for LR, EBM, and XGB models. Solid lines represent models trained on EUH data only; dashed lines represent models trained on EUH data supplemented with MIMIC-IV data. The x-axis shows the number of EUH samples used, while the y-axis displays AUROC values for readmission predictions. Vertical lines indicate the break-even points

**Fig. 17:**
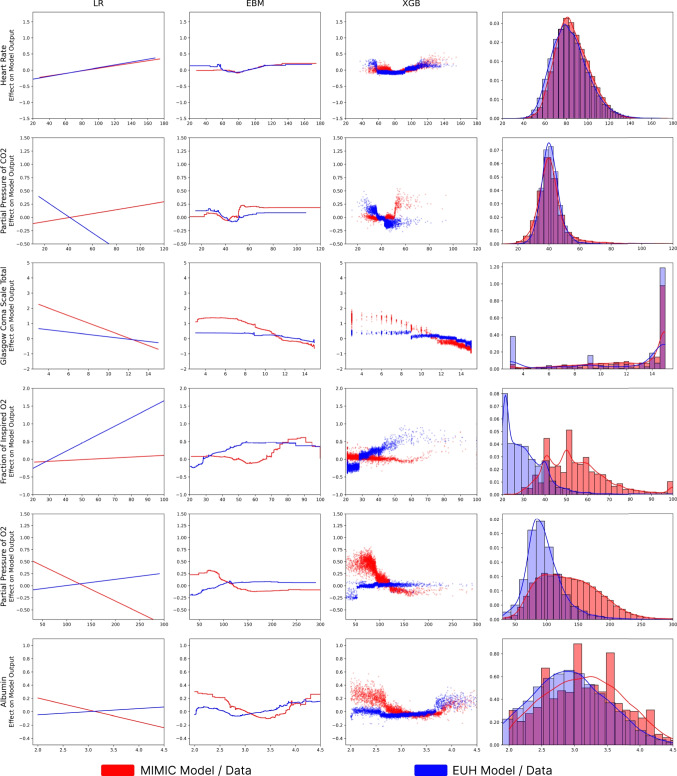
Length of stay model insights

**Fig. 18:**
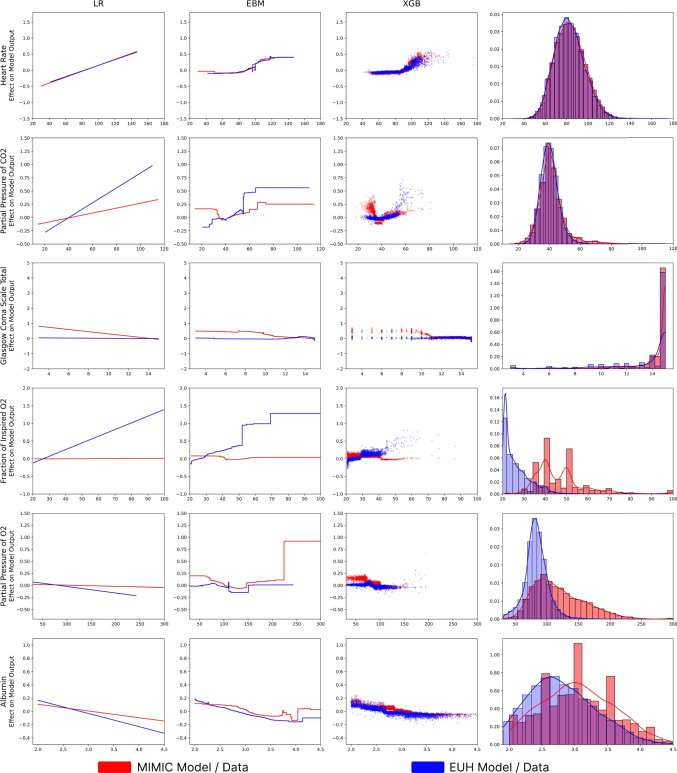
Readmission model insights

**Fig. 19:**
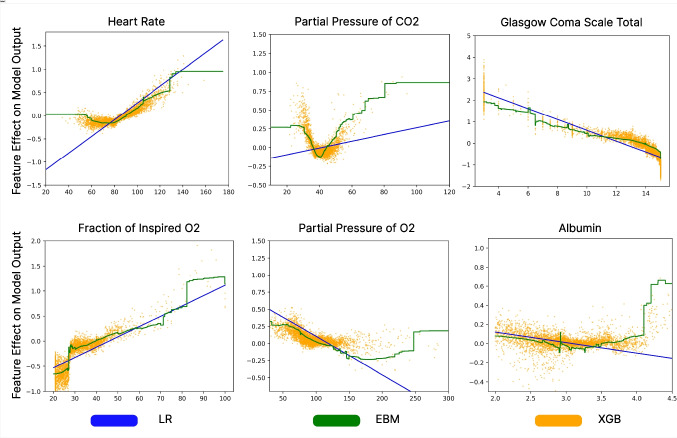
Comparison of feature-mortality relationships learned by LR, EBM, and XGB trained on the combined MIMIC-IV and EUH dataset. Plots demonstrate consistent trends across model types for each feature, indicating robust underlying relationships in the data

**Fig. 20:**
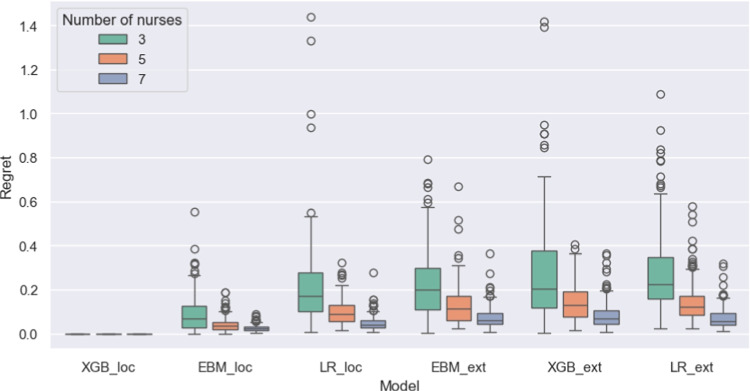
Boxplots for regret *r* for the three instance sizes

**Fig. 21:**
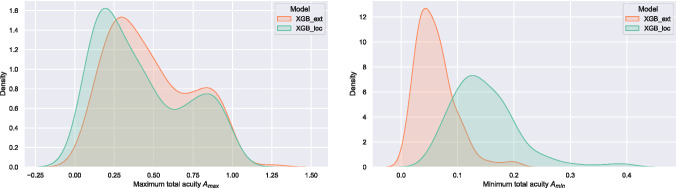
Kernel density estimate plot illustrating the distribution of $A_{max}$ and $A_{min}$ for $\mathrm{XGB\_ext}$ and $\mathrm{XGB\_loc}$ as acuity estimate input models

## Data Availability

This study utilized two datasets: 1. **MIMIC-IV (Medical Information Mart for Intensive Care IV) Database** The MIMIC-IV dataset (https://doi.org/10.13026/6mm1-ek67) is publicly available and can be accessed upon reasonable request to the PhysioNet repository after completing the required data usage agreements and training. Further details on accessing this database are available at https://physionet.org/content/mimiciv/. The database consists of de-identified health data from patients admitted to ICUs at the Beth Israel Deaconess Medical Center, Boston, Massachusetts, USA. 2. **Proprietary Dataset from Universitätsklinikum Carl Gustav Carus** The second dataset used in this study is proprietary and was obtained from "Universitätsklinikum Carl Gustav Carus" in Dresden, Germany. Due to data protection regulations, these patient-related data cannot be made publicly available. The use of the dataset containing historical, pseudonymous patient data for this study has been approved by the Ethics Committee at Technische Universität Dresden (IRB00001473) under reference number BO-EK-79022022.

## Appendix A Data and preprocessing details

### A.1 Data preprocessing steps

This appendix describes the preprocessing steps in more detail. These steps include procedures to align both datasets and other standard techniques in ML.

After loading the two datasets into a shared computing environment, we applied well-established preprocessing steps. Minor variations of these steps were necessary for each dataset to ensure a consistent final data format across both sets. Specifically, the following steps were applied:

1. **Unit Matching:** To ensure consistency and comparability between the datasets, we adjusted the units of measurement of several features from the EUH dataset to match those used in the MIMIC-IV dataset. For example, glucose measurements were converted from mmol/L to mg/dL and partial pressure of O2 measurements were transformed from kPa to mmHg.
2. **MIMIC-IV Anonymization Matching:** We apply a maximum age of 90 to patients from the European hospital dataset to match the anonymization in the MIMIC-IV dataset. Additionally, we remove all patients under 18 from the experiments since they are not included in the MIMIC-IV database.
3. **Outlier Removal:** Using the time series data, we removed unrealistic individual measurements before applying mean aggregation. We provide a detailed description of the exact lower and upper bounds in Appendix A (A.4).
4. **Mean Aggregation:** To create static features, we calculated the mean of the values from the first (mortality and length of stay) or last (readmission) 24 hours after ICU admission.
5. **Filter Patients:** Next, we excluded patients with more than 50% missing data. For the readmission task, deceased patients were also removed.
6. **Scaling and Encoding:** We standardize all numerical features to have a mean of zero and a standard deviation of one. Additionally, we apply one-hot encoding to transform categorical features, specifically gender.
7. **Missing Values Imputation:** We impute the remaining samples’ missing values using a k-nearest neighbor (KNN) imputation algorithm. This method was chosen over mean or median imputation due to its ability to capture interactions between features and account for the non-random nature of missing data in both ICU datasets.

### A.2 Data summary readmission

The feature extraction process for the readmission prediction task differs slightly from that for the other prediction targets. Specifically, clinical data from the last 24 hours of the ICU stay are used, rather than from the first 24 hours, as is done for mortality and length of stay prediction. This reflects the fact that readmission risk is most relevant at the time of discharge. Consistent with this change, deceased patients are excluded from the readmission task, since they are not eligible for discharge and cannot, by definition, be readmitted. Consequently, some descriptive statistics and sample counts differ from those presented for other prediction targets. Additionally, we have included the length of stay variable in the feature set for readmission prediction. In this context, length of stay represents the actual length of the patient’s ICU stay at discharge, which provides valuable context for estimating readmission risk. Previous studies have identified length of stay as a meaningful predictor of readmission, likely due to its association with illness severity, recovery trajectory and discharge timing decisions. Summary statistics for all features used in the readmission task are provided in Table 4, including mean, standard deviation, and missing value percentages for both the EUH and MIMIC datasets based on the last 24 hours of each ICU stay.

**Table 4:** Comparison of MIMIC and EUH datasets for the readmission prediction task. The table presents mean values, standard deviations, and missing percentages for all features

Feature	MIMIC			EUH		
	Mean	Std	Missing (%)	Mean	Std	Missing (%)
Length of Stay (h)	95.04	117.61	0.00	172.96	238.76	0.00
Weight	82.90	23.50	2.43	78.15	19.47	40.10
Age	63.28	16.40	0.00	63.15	16.73	0.01
Temperature	36.82	0.36	0.27	36.89	0.62	3.58
Respiratory Rate	19.32	3.60	0.34	18.70	4.07	43.88
Heart Rate	83.29	13.96	0.04	82.58	13.75	0.28
Glucose	125.73	40.73	1.05	131.89	27.68	0.05
Mean Blood Pressure	79.92	11.35	0.20	89.24	13.24	18.04
Potential Hydrogen	7.40	0.06	76.75	7.45	0.04	51.53
Glasgow Coma Scale Total	14.24	1.54	0.16	13.53	2.75	58.41
Gender (Female%)	42.97	-	0.00	38.94	-	0.00
Partial Pressure of O2	119.63	41.24	88.59	83.37	18.10	0.07
Creatinine	1.31	1.39	0.71	0.93	0.90	1.25
Fraction of Inspired O2	47.05	12.88	78.07	26.37	6.62	1.73
Potassium	4.05	0.48	0.69	4.13	0.33	0.10
Sodium	138.54	4.38	0.67	138.21	4.34	0.21
Leukocytes	10.53	6.20	1.83	10.21	4.59	0.96
Thrombocytes (Platelets)	212.13	118.14	1.55	256.27	138.91	1.16
Bilirubin	2.42	4.93	76.85	0.80	1.38	21.16
Bicarbonate	25.31	4.39	0.80	27.15	3.20	0.11
Lactate	1.63	0.88	86.38	1.01	0.47	0.05
Hemoglobin	10.13	1.88	1.41	9.89	1.55	0.00
Prothrombin Time	1.42	0.58	37.47	1.29	0.27	1.51
Partial Pressure of CO2	41.67	8.95	88.51	40.19	5.92	0.07
Alanine Aminotransferase	89.30	190.44	76.93	53.85	101.60	23.34
Aspartate Aminotransferase	93.71	178.29	76.84	55.86	94.54	18.68
Albumin	3.06	0.56	89.51	2.84	0.50	29.63
Anion Gap	13.11	3.08	1.13	5.46	2.17	71.11
Urea Nitrogen	24.74	19.91	0.75	40.04	29.09	12.38
Readmission 72h (%)	6.50	–	0.00	7.80	–	0.00

### A.3 Feature explanations

In this appendix, we provide detailed explanations of the medical features used in our study. Understanding the clinical relevance of each feature is critical for interpreting the results of our ML models and for facilitating replication in future research. Table 5 provides brief explanations of each feature, highlighting its clinical significance and relevance to ICU outcomes. This information clarifies how each feature contributes to the models and emphasizes the importance of certain physiological and biochemical parameters in patient prognosis.

**Table 5:** Explanation of medical features and their implications

Feature	Explanation
Age	Age of the patient, a key demographic factor influencing health outcomes.
Weight	Patient’s body weight, important for dosage calculations and overall health assessment.
Temperature	Body temperature indicates the presence of infection, inflammation, or other health conditions.
Respiratory Rate	Measures the number of breaths per minute, indicating respiratory function and distress.
Heart Rate	Measures the number of heartbeats per minute, reflecting cardiovascular health and stress.
Glucose	Indicates blood sugar levels, crucial for diagnosing and managing diabetes and metabolic health.
Mean Blood Pressure	Measures the average pressure in a patient’s arteries during one cardiac cycle, reflecting cardiovascular health.
Potential Hydrogen	Measures the acidity or alkalinity of the blood, indicating metabolic and respiratory function.
Glasgow Coma Scale Total	Assesses a patient’s level of consciousness, providing information on neurological function.
Gender	Patient’s gender, which can influence various health outcomes and treatment responses.
Partial Pressure of O2	Reflects the patient’s oxygenation status and helps assess the severity of respiratory failure.
Fraction of Inspired O2	Measures the O2 concentration delivered to the patient through ventilation, indicating respiratory support.
Potassium	Plays a fundamental role in cellular function and cardiovascular health.
Sodium	Has a crucial role in various physiological processes and reflects underlying health conditions.
Leukocytes	White blood cells, indicating immune function, inflammation, and overall health status.
Thrombocytes (Platelets)	Play a crucial role in blood clotting and wound healing; associated with sepsis outcomes, cancer, and cardiovascular diseases.
Bilirubin	Byproduct of heme catabolism in the liver; provides information about liver health and sepsis outcomes.
Bicarbonate	Key component of the body’s buffer system for pH regulation; associated with mortality outcomes.
Hemoglobin	Crucial for O2 transport throughout the body; related to all-cause mortality.
Prothrombin Time	Provides information about blood clotting functionality; related to impairments in liver function.
Alanine Aminotransferase	Enzyme primarily found in liver cells; crucial for amino acid metabolism.
Aspartate Aminotransferase	Enzyme primarily found in liver cells; crucial for amino acid metabolism.
Partial Pressure of CO2	Provides information about respiratory function and pH balance.
Albumin	Key role in physiology in the blood vessels for regulating osmotic pressure.
Anion Gap	Measures the difference between positively and negatively charged ions; helps assess acid-base balance and underlying health conditions.
Lactate	Closely related to cellular respiration oxygenation process; influences energy production and related to sepsis outcomes and overall metabolic function.
Urea Nitrogen	Byproduct of protein metabolism; associated with adverse health outcomes for various diseases.
Creatinine	A by-product of muscle metabolism, filtered by the kidneys and used to assess renal function. Elevated levels can indicate kidney impairment or dehydration.

### A.4 Feature limits

Table 6 outlines the upper and lower bounds set during the data preprocessing. These limits help prevent the inclusion of unrealistic or outlier values before the mean aggregation, ensuring that the models are trained on data within plausible ranges for clinical use.

**Table 6:** Upper and lower bounds for the medical features were applied to both datasets during feature extraction to ensure consistency

Feature	Lower Bound	Upper Bound	Unit
Weight	20	500	kg
Temperature	20	45	${}^\circ$C
Respiratory Rate	5	50	breath/min
Heart Rate	10	300	beats/min
Glucose	5	2000	mg/dl
Mean Blood Pressure	20	400	mmHg
Potential Hydrogen	5	9	–
Glasgow Coma Scale	3	15	score
Gender	-	-	-
Partial Pressure of O2	10	300	mmHg
Fraction of Inspired O2	20	100	%
Potassium	2.5	7	mmol/l
Sodium	120	160	mmol/l
Leukocytes	1	200	10³/$\mu$l
Thrombocytes (Platelets)	10	1000	10³/$\mu$l
Bilirubin	0.1	50	mg/dl
Bicarbonate	10	45	mmol/l
Hemoglobin	10	20	g/dl
Prothrombin Time (INR)	0.2	6	-
Alanine Aminotransferase	2	2000	U/l
Aspartate Aminotransferase	2	2000	U/l
Partial Pressure of CO2	10	300	mmHg
Albumin	2	6	g/dl
Anion Gap	1	25	mmol/l
Lactate	0.1	200	mmol/l
Urea Nitrogen	1	100	mg/dl
Creatinine	0.1	20	mg/dl

## Appendix B Convex hull analysis

The summary of our convex hull analysis, presented in Tables 7 and 8 provide strong theoretical support for good cross-hospital generalizability (seen in Section 4) from the perspective of feature ranges. With a minimum coverage of over 90%, even for identified problematic features, previous research suggests that ML models should be able to generalize well without facing substantial conceptual difficulties from extrapolation [72]. When analyzing the full feature space using Principal Component Analysis (PCA), we observe remarkably high and symmetric overlap between datasets: 99.96% of MIMIC test points fall within the EUH-defined convex hull as seen in Fig. 8, while 99.35% of EUH test points fall within the MIMIC-defined hull seen in Fig. 9. This near-complete bidirectional coverage indicates that the overall clinical feature spaces between hospitals are highly compatible, with most predictions representing interpolation rather than extrapolation scenarios. Despite the dimensionality reduction inherent in PCA, these findings suggest that external models should be able to maintain high predictive performance when transferred, which aligns with the cross-hospital results observed in Section 4.1.

**Table 7:** Convex hull analysis using two critical features (fraction of inspired O2, albumin)

Direction	Inside Hull	Outside Hull
EUH hull $\rightarrow$ MIMIC test	99.61%	0.39%
MIMIC hull $\rightarrow$ EUH test	90.25%	9.75%

**Table 8:** Convex hull analysis using PCA with two principal components

Direction	Inside Hull	Outside Hull
EUH hull $\rightarrow$ MIMIC test	99.96%	0.04%
MIMIC hull $\rightarrow$ EUH test	99.35%	0.65%

When examining two specific features individually, that lead to very different patterns learned by the models across datasets, we find more nuanced patterns that further complement our generalizability findings. For the two critical features (fraction of inspired O2, albumin), the convex hull overlap remains substantial but shows asymmetry: while 99.61% of MIMIC test points fall within the EUH-defined hull seen in Fig. 10, only 90.25% of EUH test points fall within the MIMIC-defined hull as seen in Fig. 11. This difference aligns with the distributional patterns identified in our interpretability analysis (Fig. 7), where these features exhibit more hospital-specific feature outcomes relationships. Importantly, even this reduced overlap of 90.25% still represents strong coverage. These findings demonstrate that from a dataset quality perspective, measured by the overlap of feature ranges, the conditions for successful model generalizability are well satisfied, with the full feature space analysis confirming that both hospitals capture fundamentally similar clinical populations despite minor variations in specific measurements. Therefore, it is reasonable to assume that the observed pattern of generalizability cannot be explained by differences in features alone.

## Appendix C Machine learning models

In this appendix, we present the six ML models used in our study, grouped by their level of complexity: low (LR and DT), medium (EBM and IGANN), and high (XGB and MLP). We provide information on the used python libraries and hyperparameter grids.

### C.1 Hyperparameter grids

We performed a systematic grid search to explore the hyperparameter space of each model to ensure they were tuned for optimal performance in each scenario we tested. This grid search was performed using cross-validation on the respective train sets. We selected the most effective hyperparameter combination for each model based on the average AUROC achieved across all train folds. It is important to note that the optimal hyperparameters found can vary depending on the task and data configuration tested. Table 9 lists the hyperparameters and corresponding ranges that we tested during the grid search process.

**Table 9:** Grid of tested hyperparameter combinations for each model

**Model**	**Tuning Parameters**	Tuning Range
LR	C (Regularization)	0.001, 0.01, 0.1, 1, 10, 100, 1000
	Penalty Term	l1, l2, elasticnet, none
	Solver	lbfgs, liblinear, saga
	Class Weight	balanced, none
	L1 Ratio	0.25, 0.5, 0.75, none
	Max Iterations	100, 300
DT	Max Depth	5, 10, 20, 40, none
	Max Leaf Nodes	none, 5, 10, 20, 40
	Class Weight	balanced, none
	Splitter	best, random
MLP	Hidden Layer Sizes	[50], [100], [25, 25], [50, 50], [75, 75], [100, 100], [25, 25, 25], [50, 50, 50], [75, 75, 75], [100, 100, 100], [50, 50, 50, 50], [100, 100, 100, 100]
	Alpha	0.0001, 0.001, 0.01
	Activation	relu, tanh
XGB	Max Depth	3, 6, 9, 12, none
	Learning Rate	0.01, 0.1, 0.3
	Number of Estimators	50, 100, 200, 500, 1000, 2000
EBM	Max Bins	256, 512
	Outer Bags	8, 16
	Inner Bags	0, 4
	Interactions	0
IGANN	Boost Rate	0.025, 0.1
	ELM Scaling Parameter	1, 2, 5
	Number of Estimators	1000, 2000, 5000
	Interactions	0

### C.2 Python implementations

All models are trained and evaluated in Python using the following libraries and versions:

- **Logistic Regression**, **Decision Tree**, and **Multilayer Perceptron**: scikit-learn (v1.6.1) — https://scikit-learn.org/
- **XGBoost (XGB)**: xgboost (v3.0.0) — https://github.com/dmlc/xgboost
- **Explainable Boosting Machine (EBM)**: interpretML (v0.6.9) — https://github.com/interpretml/interpret
- **Interpretable Generalized Additive Neural Networks (IGANN)**: igann (v0.1.7) — https://github.com/MathiasKraus/igann

## Appendix D Statistical evaluation

### D.1 Further methodological details

To complement the statistical approach described in Section 3.6, we provide some further details on the ranking procedure used for the Friedman tests with subsequent Nemenyi post-hoc tests: For each of the 25 combinations of the 5 train- and 5 test-folds, models are ranked from 1 (best) to 6 (worst), since we have six models in this comparative assessment, based on their generalizability loss. Either comparative generalizability loss (performance relative to the best tested model) or model-specific generalizability loss (absolute performance difference in external vs. local settings per model) is used resulting in two statistical analyses. The ranks across all folds serve as the test statistic for the Friedman test and the subsequent Nemenyi post-hoc test, with lower average ranks indicating superior cross-hospital generalizability.

### D.2 Average ranks

Table 10 and Table 11 present the average rankings in tabular format, corresponding to the visual and statistical assessment in Fig. 4. The comparative rankings (Table 10) show how models perform relative to the best transferred model, while the specific rankings (Table 11) capture how much each model degrades compared to itself when tested in local versus external hospital scenarios. Lower average rank values in both tables indicate better generalizability across hospital transfers.

**Table 10:** Average comparative generalizability loss ranks. Lower ranks are better and best model is marked in bold

Model	LR	DT	EBM	IGANN	XGB	MLP
Mortality	2.94	5.48	**1.00**	3.20	2.86	5.52
Length of Stay	**1.34**	4.60	2.20	2.76	4.28	5.82
Readmission	**1.68**	5.18	2.28	2.86	3.24	5.76

**Table 11:** Average model-specific generalizability loss ranks. Lower ranks are better and best model is marked in bold.

Model	LR	DT	EBM	IGANN	XGB	MLP
Mortality	**1.46**	3.88	2.12	3.54	4.66	5.34
Length of Stay	**1.24**	2.50	3.06	3.58	5.14	5.48
Readmission	**1.42**	3.42	3.98	3.10	4.52	4.56

## Appendix E Supplementary analysis of length of stay and readmission results

### E.1 Levels of local data availability

In this section, we present additional results from our low-data simulation experiments, specifically focusing on length of stay and readmission prediction tasks. While the main manuscript centers on mortality prediction due to space and clarity considerations, the same experimental approach was applied across all outcomes. These supplementary analyses provide further insight into model behavior under data-scarce conditions and demonstrate the generalizability of our findings across different clinical prediction tasks.

Figure 12 repeats the mortality analysis presented in Figure 5. Figures 13 and 14 extend these mortality results to length-of-stay and readmission predictions, respectively. The same trends observed for mortality hold across these tasks: purely local models quickly benefit from additional local samples, with simpler models gaining an early advantage, while more complex models continue to improve with larger datasets and eventually become competitive. Models initialized with external data also improve as local data are added, but at a slower rate. Across all models and tasks, we observe clear break-even points where a purely local model surpasses its externally supplemented counterpart. For length of stay prediction, these break-even points occur with only 250 samples for LR, 500 for EBM, and around 4500 for XGB (Fig. 15). For readmission prediction, the corresponding thresholds are much higher: 2250 samples for LR, 2500 for EBM, and about 8500 for XGB (Fig. 16). These results illustrate how increasing model complexity requires progressively more local data to outperform models trained with external data.

### E.2 Model insights for length of stay and readmission

In addition to the feature–outcome relationships examined for mortality predictions, we also evaluated the same models and features for the length of stay (Fig. 17) and readmission (Fig. 18) tasks using interpretability analyses. Overall, the learned relationships were largely similar to those observed for mortality, although some notable differences emerged. For example, the Glasgow Coma Scale did not appear to be a meaningful predictor of readmission. This finding is intuitive, as patients who are not fully conscious are unlikely to be discharged from the ICU and therefore rarely contribute to readmission cases. Another interesting observation was that the partial pressure of CO2 demonstrated a consistent contribution across datasets for length of stay predictions.

## Appendix F Models trained on combined dataset

We also evaluated models that were trained using a combination of the two datasets. As these models have access to all the data from both hospitals, they are expected to perform well on both test sets. Table 12 shows their performance on the three tasks for both test sets. Compared to the results in Table 3, the performance of these combined models is worse than that of the local models on their respective test sets, but better than that of the transferred models. This suggests that, while not optimal, combined models are a good solution if the aim is to deploy a single model that works across multiple hospitals.

**Table 12:** Performance of models trained on the combined dataset and tested on EUH and MIMIC-IV for mortality, length of stay, and readmission tasks

Train Set	Model	Mortality		Length of stay		Readmission	
		EUH	MIMIC-IV	EUH	MIMIC-IV	EUH	MIMIC-IV
Combination (N = 72101)	LR	83.81 ± 0.94	85.69 ± 0.33	66.41 ± 0.36	76.24 ± 0.43	60.36 ± 0.56	62.40 ± 0.65
	DT	80.79 ± 1.12	82.01 ± 0.64	65.13 ± 1.59	74.26 ± 0.63	56.54 ± 0.99	59.25 ± 0.72
	IGANN	86.78 ± 0.71	87.06 ± 0.30	68.78 ± 0.62	77.11 ± 0.39	63.10 ± 0.85	62.84 ± 0.56
	EBM	88.05 ± 0.64	87.25 ± 0.28	68.61 ± 0.82	77.12 ± 0.33	62.69 ± 0.71	63.53 ± 0.46
	XGB	88.06 ± 0.69	88.40 ± 0.33	70.85 ± 0.59	78.38 ± 0.37	62.37 ± 0.92	64.12 ± 0.51
	MLP	85.42 ± 1.00	86.55 ± 0.57	68.80 ± 0.79	77.28 ± 0.35	58.90 ± 1.47	59.00 ± 1.14

Figure 19 provides a complementary perspective, showcasing the consistency of different model types (LR, EBM, and XGB) when trained on the same combined dataset to predict mortality. Despite their varying complexities, these models extract remarkably similar trends for each feature. This consistency suggests that the underlying relationships in the data are robust enough to be captured by diverse modeling approaches. Notably, the strong performance of these combined models across both test sets and tasks highlights their potential for generalizability. Integrating diverse data sources may help in developing more broadly applicable models. However, as our feature-level analysis indicates, it is essential to account for the specific characteristics of each dataset to ensure reliable model performance in varied clinical settings.

## Appendix G Numerical study on the effect of mortality prediction on the nurse assignment problem

To further illustrate the effect a poorly generalizing model may have on clinical decision making, we conduct a small numerical study on the nurse assignment problem (NAP). The acuity-based NAP is listed in Section 2.1 as an adjacent resource allocation problem effected by patients mortality risk. In the NAP literature, patient acuity is the predominant indicator for the required workload, assuming that more acute patients are monitored more frequently or require special treatments. Since mortality risk can be used as a proxy for patient acuity [26], the acuity-based NAP fits our purpose of analyzing the impact of ML-based mortality prediction on clinical decision making.

We base our analysis on the mixed-integer program (MIP) Model 1 from [88], which aims at comparing different nursing workload indicators and fairness objectives for a simple NAP. The baseline MIP consists of few constraints concerned with minimum and maximum total patient acuity. We chose this MIP formulation to minder overlap effects with other factors (e.g. availability, qualification, etc.), that are present in more complex NAPs [29, 30], to single-out the effects of the patient acuity / mortality risk assessment.

### G.1 Problem description and MIP formulation of the NAP

The NAP is concerned with assigning *n* available nurses with the care of *p* patients, aiming at a balanced resulting workload. Each patient *p* has an acuity score $a_p$, the values of which are provided by our mortality prediction models, with $a_p \in [0,1]$. Patients with high risk of mortality are thereby associated with high acuity. Since acuity scores have a linear relation to the objective of the MIP, it is not necessary to transform the model output to the scale used in [88]. The sum of all acuity scores of the assigned patients represents the total workload of a nurse. The objective function minimizes the largest difference in total assigned acuity score within the set of nurses (G1).

The following MIP formulation, the notation of which is given in Table 13, represents the NAP:

G1 $$\begin{aligned} \mathrm{Minimize} \quad Z = A_{max} - A_{min} \end{aligned}$$

G2 $$\begin{aligned} \sum_{n=1}^{\mathcal{N}} x_{np} = 1 \quad \forall p \in \mathcal{P} \end{aligned}$$

G3 $$\begin{aligned} \sum_{p=1}^{\mathcal{P}} a_p \cdot x_{np} &\leq A_{max} && \forall n \in \mathcal{N} \end{aligned}$$

G4 $$\begin{aligned} \sum_{p=1}^{\mathcal{P}} a_p \cdot x_{np} &\geq A_{min} && \forall n \in \mathcal{N} \end{aligned}$$

G5 $$\begin{aligned} x_{np} \in \{0,1\} \quad \forall n \in \mathcal{N}, \forall p \in \mathcal{P} \end{aligned}$$

Constraints (G2) ensure the assignment of exactly one nurse to each patient. Constraints (G3) and (G4) set the values for the maximum and minimum total assigned acuity across the set of nurses. The decision variable is defined by (G5).

**Table 13:** Notation NAP

Sets and indices	
$\mathcal {N} = \{1,...,n\}$	Set of nurses
$\mathcal {P} = \{1,...,p\}$	Set of patients
Parameters	
$a_{p}$	Acuity of patient *p*
Decision variables	
$A_{max}$	Maximum total acuity assigned to a nurse
$A_{min}$	Minimum total acuity assigned to a nurse
*Z*	Span between maximum and minimum total acuity assigned
$x_{np}$	1, if nurse *n* is assigned to patient *p*; else 0

### G.2 Analysis of model impact

To assess the impact of mortality prediction models on the acuity-based nurse assignment, we solve the NAP for six different mortality prediction models and compare the resulting assignments. We focus on the most promising model variants: LR, EBM, XGB, each trained on either full local EUH train data or external MIMIC train sets. We denote the acuity scores derived from the mortality risk predicted by model $m$ as $a_p^{m}$. The set of models $\mathcal {M}$ includes both local and external versions of each model type and is defined as

G6 $$\begin{aligned} \mathcal {M} = \left\{ \begin{array}{ccc} \text {LR}_{\text {loc}}, & \text {EBM}_{\text {loc}}, & \text {XGB}_{\text {loc}}, \\ \text {LR}_{\text {ext}}, & \text {EBM}_{\text {ext}}, & \text {XGB}_{\text {ext}} \end{array} \right\} \end{aligned}$$

Other input parameters for the NAP are the number of patients and nurses, indicating the size of the ICU. We solve the NAP for $p = 20$ number of patients, which is consistent with ICU size at the university hospital, and varying number of nurses with $n \in \{ 3,5,7\}$. As there are 4047 patients in the EUH test set, we construct 202 randomly selected sets of patients, resulting in 606 total instances for each input model. The instances are solved to optimality using Gurobi 12.0.2.

Assessing which prediction model inputs lead to a better nurse assignment is difficult, since true acuity scores are unknown. We assume, that the best performing mortality prediction model, in our case the XGB model trained on local EUH data ($\text {XGB}_{\text {loc}}$), comes closest to a true acuity score and set this model as our baseline, with corresponding acuity scores $a^{\text {base}}$ and NAP objective value $Z^{\text {base}}$. Consequently, we define the regret of a suboptimal assignment due to imprecise input data in accordance with the regret/loss functions in the predict-and-optimize literature [89, 90] as:

G7 $$\begin{aligned} r(a^m,a^{\text {base}})&= Z(x^*(a^m), a^{\text {base}}) - Z^{\text {base}} \end{aligned}$$

with $x^*$ denoting the resulting decision variable values with respect to the input $a^m$.

We then utilize the following steps for each instance:

1. Solve the NAP for the input data $a^{\text {base}}$ of the baseline model $\text {XGB}_{\text {loc}}$ $\longrightarrow$ $Z^{\text {base}}$
2. For each model $m \in \mathcal {M}, m \ne \text {XGB}_{\text {loc}}$: Solve NAP $\longrightarrow$ nurse-patient-assignment $x^*(a^m)$
3. Calculate $Z(x^*(a^m), a^{\text {base}})$ for model-specific assignment $x_{pn}^{m}$ and baseline acuity scores $a^{\text {base}}$ $\longrightarrow$ $Z(x^*(a^m), a^{\text {base}}) \ge Z^{\text {base}}$
4. Assess regret of the suboptimal assignment due to the imprecise prediction of $a^m$ using (G7)

Table 14 shows the averaged results over all 202 instances for three instance sizes, the variance between the instances is further illustrated in Fig. 20.

**Table 14:** Averaged results over the 202 instances for the three instance sizes

Instance size	Model	AUROC	Average Regret *r*
n = 3	$\text {XGB}_{\text {loc}}$	89.27	0
	$\text {EBM}_{\text {loc}}$	89.02	0.087 (±0.081)
	$\text {LR}_{\text {loc}}$	86.60	0.210 (±0.180)
	$\text {EBM}_{\text {ext}}$	81.56	0.224 (±0.152)
	$\text {XGB}_{\text {ext}}$	79.55	0.269 (±0.223)
	$\text {LR}_{\text {ext}}$	79.36	0.278 (±0.180)
n = 5	$\text {XGB}_{\text {loc}}$	89.27	0
	$\text {EBM}_{\text {loc}}$	89.02	0.041 (±0.032)
	$\text {LR}_{\text {loc}}$	86.60	0.098 (±0.054)
	$\text {EBM}_{\text {ext}}$	81.56	0.130 (±0.091)
	$\text {XGB}_{\text {ext}}$	79.55	0.146 (±0.084)
	$\text {LR}_{\text {ext}}$	79.36	0.141 (±0.091)
n = 7	$\text {XGB}_{\text {loc}}$	89.27	0
	$\text {EBM}_{\text {loc}}$	89.02	0.025 (±0.015)
	$\text {LR}_{\text {loc}}$	86.60	0.046 (±0.032)
	$\text {EBM}_{\text {ext}}$	81.56	0.073 (±0.048)
	$\text {XGB}_{\text {ext}}$	79.55	0.084 (±0.060)
	$\text {LR}_{\text {ext}}$	79.36	0.073 (±0.049)

In general, models with higher predictive performance also lead to lower regret for the subsequent nurse assignment, especially generalizability loss of external models can cause larger optimality gaps. The resulting shifts in maximum and minimum total acuity are further illustrated in Fig. 21 for the local and external XGB model and instances with $n = 5$. Both components of the objective function are affected, with $A_{max}$ shifting to the right, increasing the maximum workload assigned to a single nurse, and $A_{min}$ shifting to the left, increasing imbalance of workload within the set of nurses. In addition, a higher nurse-patient-ratio is more robust to non-optimal acuity predictions.

For exemplification, these results suggest that, in the event of limited local data, it is more advantageous to use a medium complexity model such as an EBM to inform NAP decisions than to use a more complex externally trained model such as XGB.

For some instances, the regret caused by worse performing external models (in terms of predictive performance) is lower than the regret caused by the local models. This can be attributed to the combinatorial nature of the NAP, which can result in better assignments in terms of Z even on the basis of less accurate acuity predictions, since prediction error and NAP objective are optimized independently.

## Appendix H Checklist for supervised clinical ML study

In Table 15, we provide a checklist that follows the guidelines proposed by Norgeot et al. [91]. The checklist is designed to ensure that all critical aspects of ML study design, implementation, and evaluation are properly addressed and documented. Each item has been carefully assessed in relation to our current work, with page references provided for easy verification.

**Table 15:** Checklist for supervised clinical machine learning study

Before Paper Submission	Completed	Page	Notes if not completed
Study Design (Part 1)			
The clinical problem in which the model will be employed is clearly detailed in the paper.	$\blacksquare$	Sections 1 and 2.1	
The research question is clearly stated.	$\blacksquare$	Section 1	
The characteristics of the cohorts (train and test sets) are detailed in the text.	$\blacksquare$	Section 3.3 and Table 2	
The cohorts (train and test sets) are shown to be representative of real-world clinical settings.	$\blacksquare$	Section 3.3 and Appendix B	
The state-of-the-art solution used as a baseline for comparison has been identified and detailed.	$\blacksquare$	Section 3.4	
Data and Optimization (Parts 2, 3)			
The origin of the data is described and the original format is detailed in the paper.	$\blacksquare$	Section 3.3	
Transformations of the data before it is applied to the proposed model are described.	$\blacksquare$	Section 3.3 and Appendix A	
The independence between train and test sets has been proven in the paper.	$\blacksquare$	Section 3.6	
Details on the models that were evaluated and the code developed to select the best model are provided.	$\blacksquare$	Sections 3.4 and 3.6; Appendix C	
Is the input data type structured or unstructured?	$\blacksquare$ Structured $\square$ Unstructured		
Model Performance (Part 4)			
The primary metric selected to evaluate algorithm performance (e.g., AUC, F-score, etc.) including the justification for selection, has been clearly stated.	$\blacksquare$	Section 3.6	
The primary metric selected to evaluate the clinical utility of the model (e.g., PPV, NNT, etc.) including the justification for selection, has been clearly stated.	$\square$	X	The paper doesn’t specifically discuss clinical utility metrics like PPV or NNT.
The performance comparison between baseline and proposed model is presented with the appropriate statistical significance.	$\blacksquare$	Section 4.1 and Appendix D	
Model Examination (Part 5)			
Examination Technique 1: Feature effect visualization/analysis	$\blacksquare$	Section 4.3	
Examination Technique 2: Break-even point analysis	$\blacksquare$	Section 4.2	
A discussion of the relevance of the examination results with respect to model/algorithm performance is presented.	$\blacksquare$	Sections 4.3 and 5	
A discussion of the feasibility and significance of model interpretability at the case level if examination methods are uninterpretable is presented.	$\blacksquare$	Sections 4.3 and 5	
A discussion of the reliability and robustness of the model as the underlying data distribution shifts is included.	$\blacksquare$	Sections 4.1, 4.3, and 5	
Reproducibility (Part 6): choose appropriate tier of transparency			
Tier 1: complete sharing of the code	$\blacksquare$		
Tier 2: allow a third party to evaluate the code for accuracy/fairness; share the results of this evaluation	$\square$		
Tier 3: release of a virtual machine (binary) for running the code on new data without sharing its details	$\square$		
Tier 4: no sharing	$\square$		

PPV: Positive Predictive Value

NNT: Numbers Needed to Treat

## Footnotes

1 Use of this dataset has been approved by the Ethics Committee at Technische Universität Dresden (IRB00001473, reference BO-EK-79022022).

2 As an alternative approach to cross-hospital deployment, we also evaluated models trained on combined data from both hospitals. These results, along with the corresponding interpretability analysis, are presented in Appendix F.

## Code Availability

All code is available under: https://github.com/HB-Dynamite/TUDD-data-analysis.
